# Mn-single-atom nano-multizyme enabled NIR-II photoacoustically monitored, photothermally enhanced ROS storm for combined cancer therapy

**DOI:** 10.1186/s40824-023-00464-w

**Published:** 2023-12-04

**Authors:** Xiaozhe Wang, Xiaofeng Ren, Jie Yang, Zican Zhao, Xiaoyu Zhang, Fan Yang, Zheye Zhang, Peng Chen, Liping Li, Ruiping Zhang

**Affiliations:** 1 The Radiology Department of First Hospital of Shanxi Medical University, First Hospital of Shanxi Medical University, Taiyuan, 030001 China; 2 College of Medical Imaging, Shanxi Medical University, Taiyuan, 030001 China; 3 Department of Biochemistry and Molecular Biology, School of Basic Medical Sciences, Shanxi Medical University, Taiyuan, 030001 China; 4 Third Hospital of Shanxi Medical University, Shanxi Bethune Hospital, Shanxi Academy of Medical Sciences, Taiyuan, 030032 China; 5 School of Chemistry, Chemical Engineering and Biotechnology, Lee Kong Chian School of Medicine, Institute for Digital Molecular Analytics and Science, Nanyang Technological University, 62 Nanyang Drive, Singapore, 637459 Singapore

**Keywords:** Single-atom nanozymes, NIR-II photoacoustic imaging, Nanocatalytic therapy, Photothermal therapy

## Abstract

**Rationale:**

To realize imaging-guided multi-modality cancer therapy with minimal side effects remains highly challenging.

**Methods:**

We devised a bioinspired hollow nitrogen-doped carbon sphere anchored with individually dispersed Mn atoms (Mn/N-HCN) via oxidation polymerization with triton micelle as a soft template, followed by carbonization and annealing. Enzyme kinetic analysis and optical properties were performed to evaluate the imaging-guided photothermally synergized nanocatalytic therapy.

**Results:**

Simultaneously mimicking several natural enzymes, namely peroxidase (POD), catalase (CAT), oxidase (OXD), and glutathione peroxidase (GPx), this nano-multizyme is able to produce highly cytotoxic hydroxyl radical (•OH) and singlet oxygen (^1^O_2_) without external energy input through parallel and series catalytic reactions and suppress the upregulated antioxidant (glutathione) in tumor. Furthermore, NIR-II absorbing Mn/N-HCN permits photothermal therapy (PTT), enhancement of CAT activity, and photoacoustic (PA) imaging to monitor the accumulation kinetics of the nanozyme and catalytic process in situ. Both in vitro and in vivo experiments demonstrate that near-infrared-II (NIR-II) PA-imaging guided, photothermally enhanced and synergized nanocatalytic therapy is efficient to induce apoptosis of cancerous cells and eradicate tumor tissue.

**Conclusions:**

This study not only demonstrates a new method for effective cancer diagnosis and therapy but also provides new insights into designing multi-functional nanozymes.

**Graphical Abstract:**

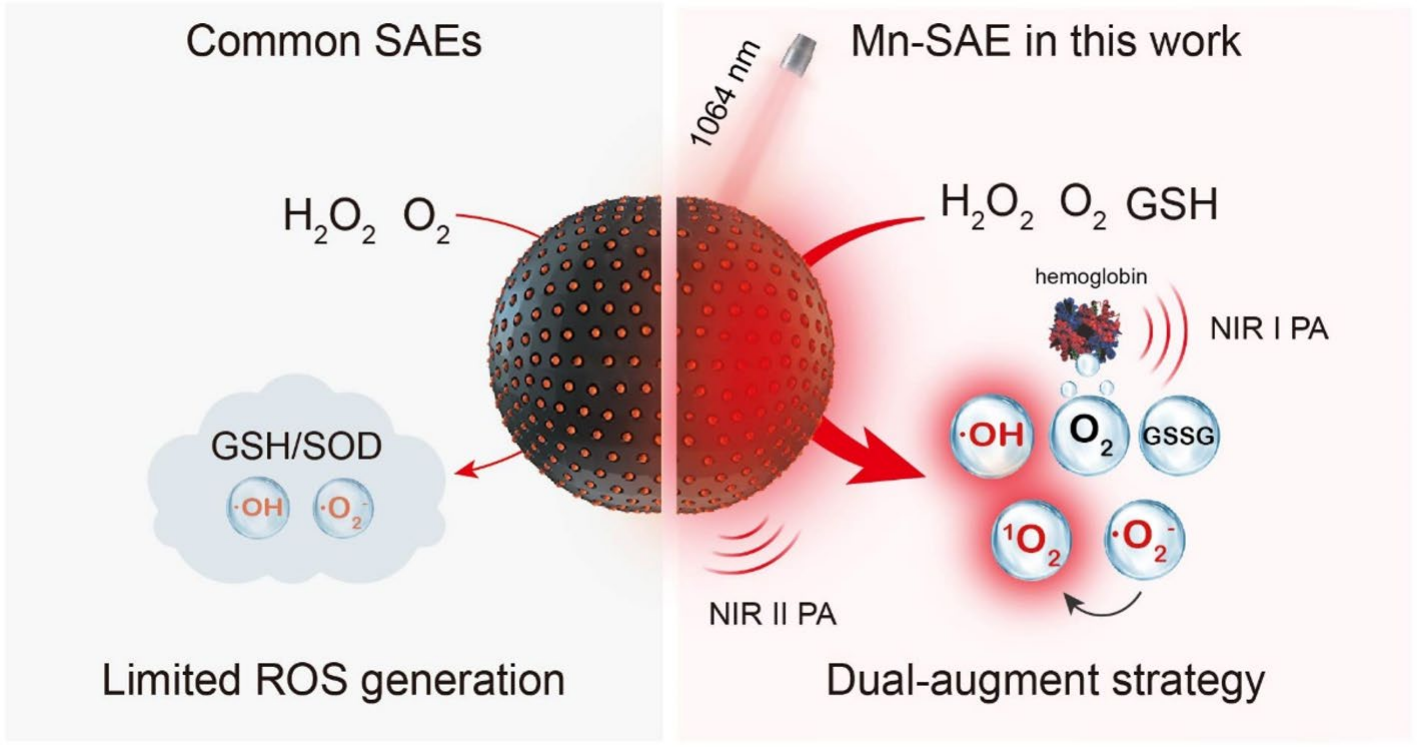

**Supplementary Information:**

The online version contains supplementary material available at 10.1186/s40824-023-00464-w.

## Introduction

Nanozymes, a kind of nanomaterials with intrinsic catalytic activities mimicking natural enzymes [1–3], have attracted considerable interest in cancer treatment [4–6]. Nanocatalytic cancer therapy is mainly based on the ability of nanozymes to produce cytotoxic reactive oxygen species (ROS) [7–9]. Compared to the conventional treatments, it offers high effectiveness, good tumor-specificity, and low side effects [10–12]. Single-atom nanozymes (SAEs) with atomically dispersed metal active sites are the most recent appealing addition to the nanozyme family because of their superior catalytic activity, highest atomic utilization, and highly tunable catalytic properties by engineering the coordination environment [13–18]. Current SAEs, however, usually lack sustainable catalytic activity, multi-enzymatic properties, and the ability to self-reveal their tumor accumulation and activity. And their effectiveness is often compromised by the upgraded antioxidant defense systems in tumor tissues [19–24]. Moreover, the readily fabrication of a biocompatible supporting material that ensures atomic dispersion, stable anchoring, and high loading of metal active sites is challenging.

Manganese (Mn) is a common co-factor of various metalloenzymes in humans [25–28]. This has recently spurred a curiosity to develop Mn-based SAEs. Li et al. reported a Mn-SAE that mimics peroxidase (POD) to catalyze H_2_O_2_ into hydroxyl radicals (•OH) [29]. The efficiency of this single-enzyme-mimicking agent is largely compromised by the limited supply of H_2_O_2_ in tumor tissues. Liu′s group demonstrated another Mn-SAE that simultaneously mimics POD, catalase (CAT) to catalyze H_2_O_2_ into O_2_, and oxidase (OXD) to convert O_2_ to superoxide radicals (•O_2_^−^) [30]. The potency of this multi-enzyme-mimicking agent is enhanced by parallel and cascaded catalytic reactions using multiple substrates. But it is undermined by overexpressed superoxide dismutase (SOD) in tumor tissues, which eliminates •O_2_^−^. Furthermore, ROS produced by both Mn-SAEs are greatly neutralized by the overexpressed antioxidants (particularly, glutathione) in tumor tissues.

**Scheme 1.**
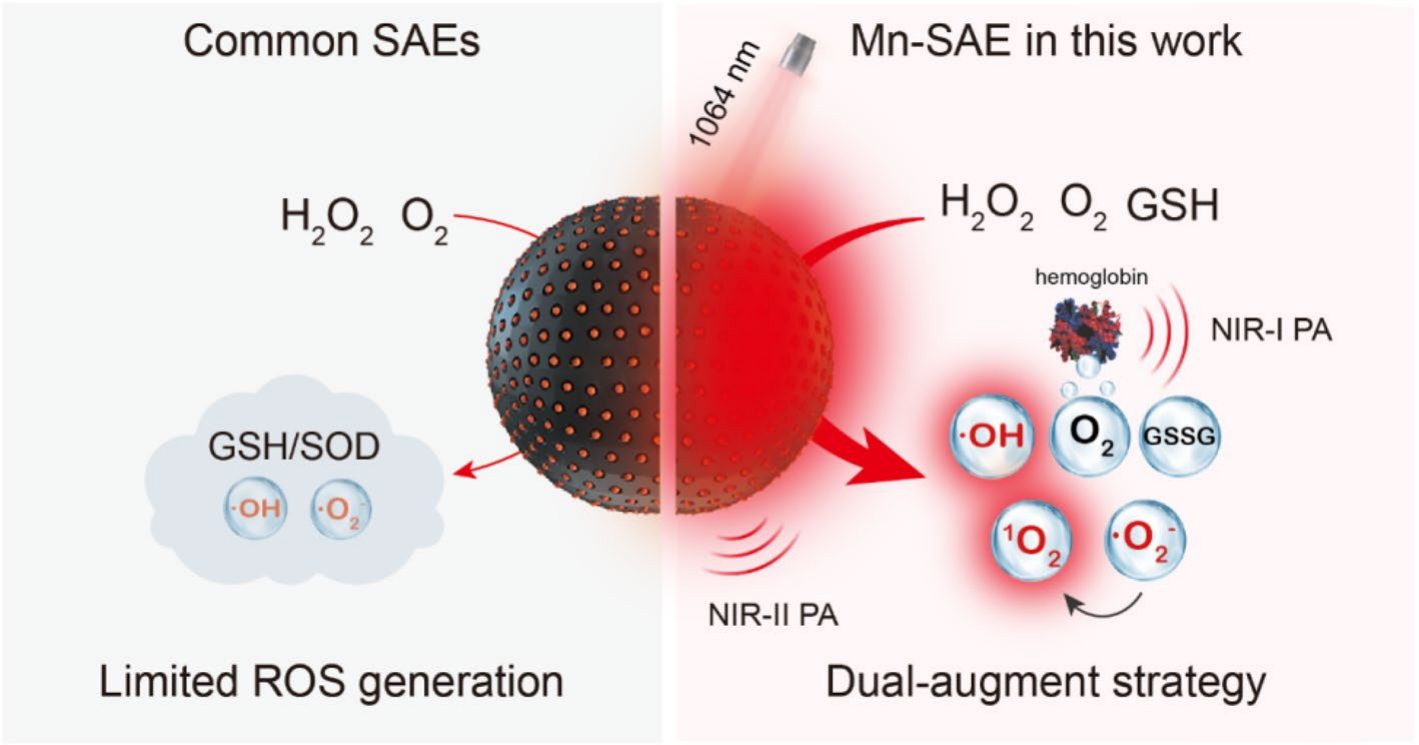
Schematic comparison between common single-atom-nanozymes (SAEs) and Mn-SAE in this work

Herein, we readily fabricated a Mn-SAE by soft-templated polymerization and carbonization. With individual Mn atoms anchored by doped nitrogen atoms (Mn-N_4_) populated on a hollow carbon nanosphere (Mn/N-HCN), this Mn-SAE simultaneously mimics POD, CAT, and OXD to produce •O_2_^−^ and highly cytotoxic •OH. Furthermore, it catalyzes •O_2_^−^ into more cytotoxic singlet oxygen (^1^O_2_), and acts as glutathione peroxidase (GPx) to eliminate GSH thereby ensuring sustainable actions by the produced ROS. In addition, with wide-spectrum adsorption, Mn/N-HCN allows NIR-II photoacoustic (PA) imaging, NIR-II photothermal therapy, and significant enhancement of the CAT-mimicking activity. Monitoring the biodistribution, accumulation kinetics in tumor tissue, and in situ catalytic activity of the nanozyme by PA imaging permits the guidance and optimization of the treatment. As shown by both in vitro and in vivo experiments, the herein demonstrated NIR-II PA-imaging guided, photothermally enhanced and synergized nanocatalytic therapy (Scheme 1) is highly efficient to induce apoptosis of cancerous cells and eradicate tumor tissue.

## Methods and materials

### Chemicals and agents

All the chemical reagents were analytical grade without further purification. Aniline, pyrrole, and ammonium persulphate were purchased from Energy Chemical (Anhui, China). 5,5′-Dithiobis(2- nitrobenzoic acid) (DTNB) was purchased from TCI (Shanghai, China). The superoxide anion assay kit was acquired from Suzhou Comin Biotechnology (Jiangsu, China). The H_2_O_2_ test kit was obtained from Nanjing Jiancheng Bioengineering Institute (Nanjing, China). The Cell Counting Kit-8 (CCK-8) kit was purchased from Sevenbio (Beijing, China). Triton X-100, calcein acetoxymethyl ester (Calcein-AM)/propidium iodide (PI) double stain kit, Annexin V-FITC Apoptosis Detection Kit, JC-1 staining kit, Acridine Orange (AO) staining kit, and 2′7′-Dichloroflfluorescein diacetate (DCFH-DA) were purchased from Beijing Solarbio Science & Technology Co., Ltd. (Beijing, China). BALB/c mice (6 weeks) were obtained from Beijing Vital River Laboratory Animal Technology Co., Ltd. VEVO LAZR instrument from FUJIFILM VisualSonics (Amsterdam, NL) operating with an LZ250 transducer was utilized for US and PA imaging.

### Characterization

Transmission electron microscopy (TEM) images, elemental mapping, and energy dispersive spectrum (EDS) analysis were observed using a JEM-2100F microscope (JEOL, Japan). The zeta potential analysis and dynamic light scattering (DLS) of the nanozyme were performed on a Nano-Zetasizer (Malvern, Zetasizer Nano ZS90). A UV–Vis-NIR spectrometer (HL-2000, TS OPTICS) was used to record the absorption spectra of nanozyme. Fourier-transform infrared (FT-IR) spectra were measured by a Vertex Perkin-Elmer 580BIR spectrophotometer (Bruker) with KBr powder as the background correction to analyze the enzyme's chemical bonds and functional groups. Thermal images were obtained using a Fluke Ti400. Thermo Scientific K-alpha instrument carried out x-ray photoelectron spectroscopy (XPS) spectra to analyze the elemental composition and the valence of the Mn component of Mn/N-HCN nanozymes. An inductively coupled plasma optical emission spectrometry (ICP-OES) (PerkinElmer 8300) was conducted for quantitative analysis of the contents of elements. X-ray absorption of fine structures (XAFS) measurements of Mn K-edge are carried out at the XAS station (BL14W1) of the Shanghai Synchrotron Radiation Facility (SSRF). The electron storage ring was operated at 3.5 GeV. Si(311) double-crystal was used as the monochromator, and the data was collected using a solid-state detector under ambient conditions. The beam size was limited by the horizontal and vertical slits with an area of 1 × 4 mm^2^ during XAS measurements.

### Synthesis of Mn/N-HCN

0.06 g of Triton X-100 was added to 60 mL of deionized water and mixed well, then 0.38 mL of aniline and 0.29 mL of pyrrole were added to form a homogeneous solution. Pre-cooled aqueous ammonium persulfate solution was added to the above pre-cooled homogeneous solution for polymerization for 12 h at 0 °C. The product was washed with deionized water until the filtrate was colorless, and the resulting product was dried at 60 °C in a drying oven to obtain the black powder. The drying powder was placed in a tube furnace and heated to 1000 °C under N_2_ flow with a heating rate of 10 °C/min for 2 h to obtain hollow carbon nanospheres (N-HCN). 50 mg N-HCN powder was dispersed in 5 mL of deionized water by ultrasonication for 5 min. After forming a homogeneous mixture, aqueous MnCl_2_·4H_2_O solution was slowly dropped into the above-mixed solution under stirring at room temperature for 3 h. The product was centrifuged, washed 3 times with deionized water, and dried in the oven at 60 °C for 24 h. The obtained powder was placed in a tube furnace and heated to 1000 °C with a heating rate of 10 °C/min for another 2 h under an N_2_ atmosphere to obtain the product Mn/N-HCN finally.

### Measurement of peroxidase-mimic activity (POD-mimic)

The POD-mimic activity of Mn/N-HCN was measured by using 3,5,3',5’-tetramethylbenzidine (TMB) as a probe. Specifically, H_2_O_2_ (25 mM) was mixed with HAc-NaAc buffer, followed by the addition of Mn/N-HCN or N-HCN (100 μg/mL and TMB (0.5 mM). The absorbance at 652 nm was recorded using a UV–Vis spectrophotometer.

The concentration dependence of the POD-mimic activity of Mn/N-HCN was assessed by adding various concentrations of Mn/N-HCN (0, 12.5, 25, 50, 100 μg/mL), H_2_O_2_ (25 mM) and TMB (0.5 mM) into HAc-NaAc buffer (pH = 4.6), the mixture was incubated for 10 min. Finally, the absorbance at 652 nm of the mixture was detected by a UV–Vis spectrophotometer.

The kinetic assays of Mn/N-HCN with H_2_O_2_ as the substrate was conducted by adding TMB (0.5 mM) and various concentrations of H_2_O_2_ solution (0, 5, 10, 20, 40, 60, 80 mM). The absorbance of productions was monitored at different reaction times.

### Measurement of catalase-like activity (CAT-mimic)

The CAT-like activity of Mn/N-HCN was detected by measuring the O_2_ production through a dissolved oxygen meter. Mn/N-HCN or N-HCN (100 μg/mL) were added into -PBS (pH 4.6 or 6.2 or 7.4) containing H_2_O_2_ (0.5 mM). The generation of O_2_ was measured at different time points. A similar method was used to detect the O_2_ production of Mn/N-HCN at 25 °C and 40 °C.

### Measurement of oxidase-like activity (OXD-mimic)

Superoxide Anion Assay Kit was used to detect •O_2_^−^. The detection was performed according to the kit steps and the concentration of Mn/N-HCN was 0, 12.5, 25, 50 and 100 μg/mL.

### Measurement of single oxygen (.^1^O_2_)

The ability of ^1^O_2_ generation by Mn/N-HCN was evaluated by a specific probe ABDA. ABDA can react with ^1^O_2_ to produce endoperoxide, which will cause the fluorescence intensity decrease of ABDA. Briefly, H_2_O_2_ (1 mM), ABDA (1 mg/mL), and Mn/N-HCN (100 μg/mL) with or without •O_2_^−^ scavenger SOD (200 units/mL) were added to the buffer (pH = 6.2). Then, the changes in fluorescence intensity were detected by a fluorescence spectrophotometer after different reaction times (0, 1, 3,5, 10, 15 min).

### Electron paramagnetic resonance (ESR) detects the reactive oxygen species

DMPO was used as the •OH and •O_2_^−^ trapping agent for the ESR assay, and TEMP was used as the ^1^O_2_ trapping agent. DMPO was mixed with Mn/N-HCN (100 μg/mL) and H_2_O_2_ (100 mM) in HAc-NaAc buffer (pH = 4.6) to detect •OH. DMPO was mixed with Mn/N-HCN (100 μg/mL) in methanol to detect •O_2_^−^. TEMP was mixed with Mn/N-HCN (100 μg/mL) and H_2_O_2_ (100 mM) in PBS (pH = 6.2) to detect ^1^O_2_. For comparison, all the groups with irradiation (1064 nm, 1.0 W/cm^2^, 5 min) were at the same condition.

### GSH depletion with Mn/N-HCN nanozymes

Mn/N-HCN (0, 50, 100, 150, 200 μg/mL) were mixed with GSH (2 mM) in PBS, then added DTNB (0.2 mM) to detect the -SH group in GSH. A UV–Vis spectrophotometer recorded the absorbance changes after a certain reaction time. The ability of Mn/N-HCN (100 μg/mL) to deplete GSH at appointed times was assessed using a similar method.

### In vitro photothermal performance evaluation

The photothermal characteristics of Mn/N-HCN with different concentrations (0, 25, 50, 100, and 200 μg/mL) under NIR-II laser irradiation (1064 nm, 1 W/cm^2^) were recorded using a thermal detector. The photothermal stability of Mn/N-HCN was evaluated by exposing it to a 1064 nm laser for 10 min, and the 1064 nm laser on/off repetition was carried out for 5 times. The photothermal conversion efficiency (*η*) was calculated as follows equation.

$$\eta =\frac{hS\left({T}_{max}-{T}_{surr}\right)-{Q}_{dis}}{I(1-{10}^{-{A}_{1064}})}$$

where *S* (cm^2^) refers to the surface area of the container; *h* (W cm^−2^ K^−1^) is the heat transfer coefficient; *T*_*max*_* (K)* is the equilibrium temperature; *T*_*surr*_ is the surrounding ambient temperature; *Q*_*dis*_* (W)* represents the heat loss due to absorption by the container which is negligible; *I (W/cm*^*2*^*)* is power intensity of the incident laser. *A*_*1064*_ means the absorbance of the Mn/N-HCN at 1064 nm.

In addition, *hS* is calculated using the following equation:

$$hS=\frac{{m}_{D}{C}_{D}}{{\tau }_{S}}$$

where *τ*_*s*_ represents the time constant of the sample system; *m*_*D*_ and C_D_ are the mass and heat capacity (4.2 J g^−1^ °C^−1^) of DI water. *τ*_*s*_is calculated using the following equations:

$${\tau }_{S}=-\frac{\mathrm{ln}\theta }{t}$$

$$\theta =\frac{T-{T}_{surr}}{{T}_{max}-{T}_{surr}}$$

where *t* is irradiation time; *θ* means the driving force temperature.

### Cell culture

The murine breast cancer line (4T1 cells) was cultured in 1640 medium containing 10% FBS and 1% penicillin–streptomycin at 37 °C under 5% CO_2_.

### Cell uptake

4T1 cells were seeded in 24-well plates and incubated for 24 h. After replacing the medium with the FITC labeled Mn/N-HCN (50 μg/mL), cells were further incubated at different times (0 h, 2 h, 4 h, 8 h, 12 h). The fluorescence imaging of cells was recorded by confocal microscopy.

### Cytotoxicity assessments

4T1 cells were seeded in 96-well plates and incubated for 24 h. The different concentrations (0, 3.12, 6.25, 12.5, 25, 50, 100 μg/mL) of Mn/N-HCN with fresh 1640 medium were added to the 96-well plate. After 10 h of incubating, the cells were irradiated with or without a 1064 nm laser (1 W/cm^2^) for 5 min. The cells were further incubated for another 2 h. The CCK-8 assay was used to detect cell viability.

### Evaluation of cell apoptosis

For analysis of cell apoptosis, Annexin V-FITC, and PI assay were employed.4T1 cells were seeded in 96-well plates and incubated for 24 h before treatment. After that, the 1640 medium was replaced with fresh acidified 1640 containing H_2_O_2_ (100 μM), Mn/N-HCN (50 μg/mL), Mn/N-HCN (50 μg/mL) with H_2_O_2_ (100 μM) for 10 h incubation. The cells treated with Mn/N-HCN with H_2_O_2_ were divided into two groups, one of which was exposed to 1064 nm laser irradiation (1 W/cm^2^) for 5 min. After another 2 h, the cells were treated according to the manufacturer's protocol of the apoptosis kit. Flow cytometry was employed to quantify apoptosis.

### Live/dead cell staining assay

For live/dead cell staining assay, 4T1 cells were seeded in 96-well plates and incubated for 24 h before treatment. Then, the 1640 medium was replaced with fresh acidified 1640 medium containing H_2_O_2_ (100 μM), Mn/N-HCN (50 μg/mL), Mn/N-HCN (50 μg/mL) with H_2_O_2_ (100 μM) and incubated for another 10 h. The cells treated with Mn/N-HCN with H_2_O_2_ were divided into two groups, one of which was irradiated with a 1064 nm laser (1 W/cm^2^) for 5 min. After another 2 h of incubation, the cells were washed with PBS three times, followed by staining with Calcein-AM and PI for 20 min. The cells were imaged by fluorescence microscopy.

### Evaluation of intracellular O_2_ generation

Intracellular oxygen production was detected using [Ru(dpp)_3_]Cl_2_ as a probe. 4T1 cells were inoculated in 96-well plates and incubated with 1640 medium for 24 h. Subsequently, the cells were incubated with 1640 medium containing 30 μM [Ru(dpp)_3_]Cl_2_ for 12 h. After that, the cells were washed with PBS three times and further incubated with 50 μg/mL Mn/N-HCN and 100 μM H_2_O_2_ for 30 min. The cells of the laser group were exposed to a 1064 nm laser irradiation (1 W/cm^2^) for 5 min. After washing with PBS for three times, the cells were imaged immediately by a fluorescence microscope.

### Detection of ROS in cells

ROS generation in cells was measured by using the Reactive Oxygen Species Assay Kit. 4T1 cells were seeded in 96-well plates and cultured with 1640 medium for 24 h. Then, acidified 1640 medium containing 50 μg/mL Mn/N-HCN (with or without 100 μM H_2_O_2_) was co-cultured with 4T1 cells for 12 h. After 10 h incubation, the cells of the laser group were exposed to a 1064 nm laser irradiation (1 W/cm^2^) for 5 min and incubated for another 2 h. Then the medium was removed, and the 1640 medium containing DCFH-DA (10 μM) was added for 15 min. After washing with PBS for three times, the cells were imaged by a fluorescence microscope.

### Analysis of lysosomal disruption

Acridine orange (AO) was used as an indicator to evaluate Mn/N-HCN-induced lysosomal damage. Briefly, 4T1 cells were seeded in 96-well plates and incubated for 24 h before treatment. After that, the 1640 medium was replaced with fresh acidified 1640 containing H_2_O_2_ (100 μM), Mn/N-HCN (50 μg/mL), Mn/N-HCN (50 μg/mL) with H_2_O_2_ (100 μM) and incubated for another 10 h. The cells treated with Mn/N-HCN with H_2_O_2_ were divided into two groups, one of which was exposed to a 1064 nm laser irradiation (1 W/cm^2^) for 5 min. After another 2 h, the cells were washed with PBS for three times and stained with AO (10 μM) for 20 min. The fluorescence imaging of cells was analyzed by fluorescence microscopy.

### Analysis of the change of mitochondrial membrane potential

Mitochondrial membrane potential was measured by the JC-1 kit. JC-1 is a fluorescent probe that could target mitochondria. The red fluorescence indicates healthy mitochondria, while the green fluorescence indicates disrupted mitochondria. 4T1 cells were seeded in 96-well plates and incubated for 24 h. After that, the 1640 medium was replaced with fresh acidified 1640 containing H_2_O_2_ (100 μM), Mn/N-HCN (50 μg/mL), Mn/N-HCN (50 μg/mL) with H_2_O_2_ (100 μM) and incubated for another 10 h. The cells treated with Mn/N-HCN with H_2_O_2_ were divided into two groups, one exposed to a 1064 nm laser irradiation (1 W/cm^2^) for 5 min. After another 2 h, the cells were washed with PBS three times and treated with a JC-1 kit according to the manufacturer's protocol. The fluorescence imaging of cells was analyzed by fluorescence microscopy.

### Western blot analysis

For western blots of Caspase-3, Bax, Bcl-2, and β-actin expressions, the cells were treated with the same methods. After incubation for 12 h, cells were harvested and lysed in RIPA lysis buffer. The total protein was obtained by centrifugation (11,000 rpm, 10 min). Then, the protein amounts were determined by using a bicinchoninic acid (BCA) protein assay kit according to the manufacturer’s instructions. The proteins were detected for a standard western blot process.

### In vivo PA imaging

PA imaging was used to monitor the injected Mn/N-HCN and the saturated vascular O_2_ (sO_2_) in the tumor site for 24 h. When the tumor volume reached 100 mm^3^, the mice were intravenously injected with Mn/N-HCN, and PA imaging was performed with a VEVO LAZR Imaging System at indicated time points (2 h, 4 h, 6 h, 8 h, 12 h, 24 h) after post-injection. 1064 nm laser irradiation (1 W/cm^2^, 10 min) enhanced catalytic performance of Mn/N-HCN in vivo was monitored by direct intratumoral injection of Mn/N-HCN (1 mg/mL, 50μL).

### In vivo antitumor assay

All animal experiments were approved by the Animal Ethics Committee of Shanxi Medical University. Female BALB/c mice were purchased from Beijing Vital River Laboratory Animal Technology Co., Ltd. The xenograft 4T1 murine breast tumor models were established through subcutaneous injection of 60 μL of 4T1 cells (1 × 10^6^) in the subcutaneous mice. The tumor-bearing mice were used for anti-tumor treatment until the tumor volume reached about 100 mm^3^. Tumor volume = (tumor length) × (tumor width)^2^/2. 4T1 tumor-bearing mice were randomly divided into four groups for the treatment of I: Control (no treatment), II: intravenous injection of PBS (200 μL) + Laser irradiation (1064 nm; 1 W/cm^2^), III: intravenous injection of Mn/N-HCN (1 mg/mL, 200 μL), IV: Intravenous injection of Mn/N-HCN + Laser irradiation. Two laser irradiation groups were irradiated for 10 min. The real-time temperature changes and infrared thermal imaging images of the mice were recorded with a thermal infrared camera. The tumor size and weight were measured and recorded by a ruler and electronic balance every day. After 14 days, the mice were euthanized, and their tumor tissues were collected and weighed. The major organs (heart, lung, liver, spleen, and kidney) and tumors were collected for H&E and TUNEL staining for further histological analysis.

### Statistical analysis

The experimental data were expressed quantitatively as mean ± standard error of the mean using Student's t-test for comparison. Differences were considered statistically significant at *p* < 0.01 (**), *p* < 0.001 (***), and *p* > 0.05 (No significance, NS). Each experiment included at least three replicates. The GraphPad Prism 8 software was used for graph plotting.

## Results and discussion

### Fabrication and characterization of Mn/N-HCN

**Fig. 1:**
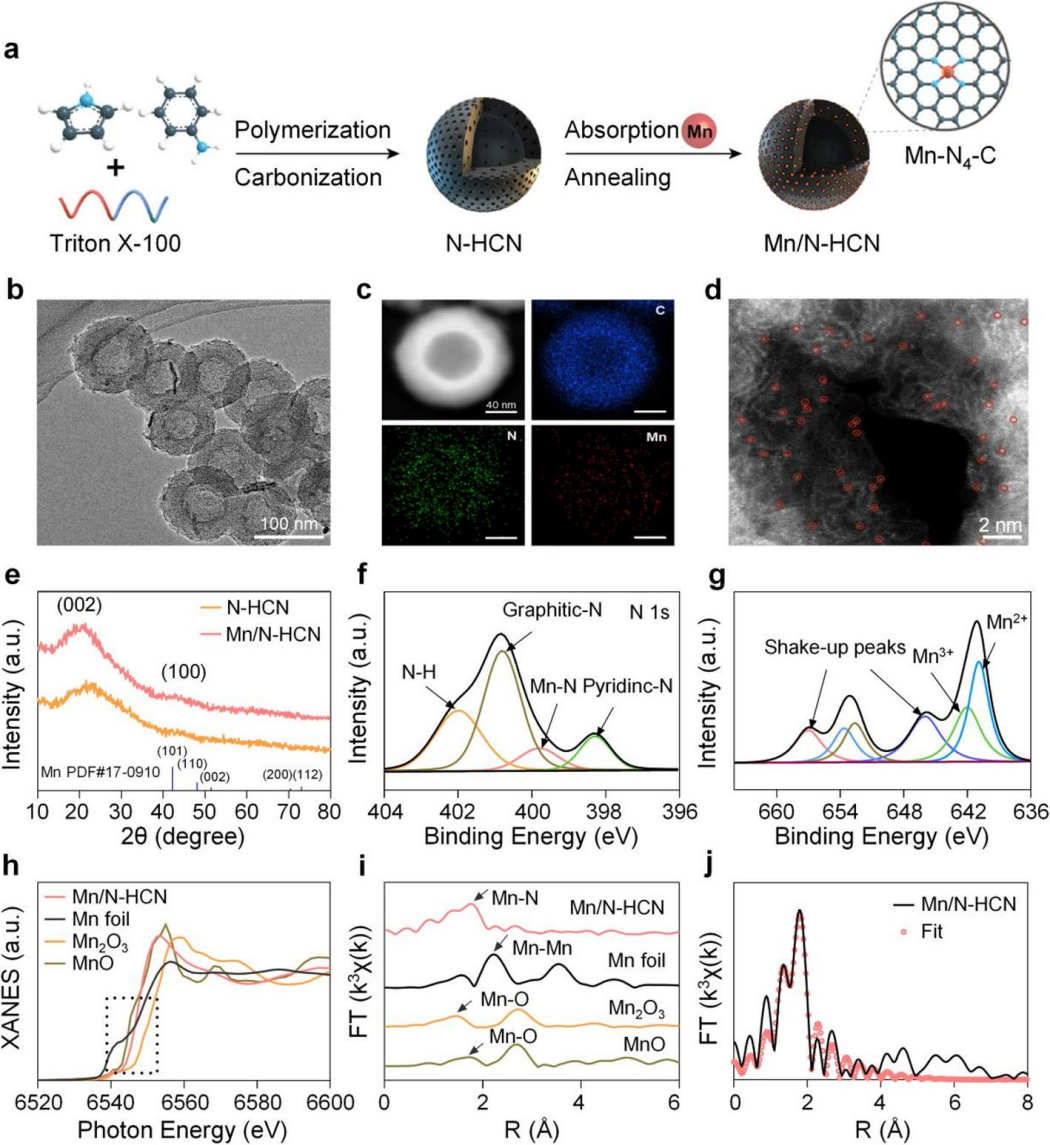
Characterizations of Mn/N-HCN. **A** Schematic illustration for the synthesis of Mn/N-HCN. **B** TEM image. **C** Energy dispersive X-ray spectroscopy elemental mapping. **D** High-magnification HAADF-STEM image where the bright dots corresponding to individual Mn atoms are highlighted by red circles. **E** XRD patterns of N-HCN and Mn/N-HCN. **F-G** High-resolution N 1 s and Mn 2p XPS spectra of Mn/N-HCN. **H-I** XANES and EXAFS spectra at the Mn K-edge. **J** EXAFS fitting curve in R space

The synthesis of Mn/N-HCN is illustrated in Fig. 1A. Firstly, nitrogen-doping hollow carbon nanospheres (N-HCN) were synthesized by the formation of poly (aniline-co-pyrrole) copolymer via oxidation polymerization with triton micelle as a soft template and subsequent carbonization (Fig. S1). Then, Mn^2+^ ions were absorbed into the highly porous N-HCN, followed by annealing to generate nanospheres decorated with individually anchored Mn atoms (Mn/N-HCN). As revealed by transmission electron microscopy (TEM) and scanning electron microscope (SEM), Mn/N-HCN display hollow spherical morphology with an average diameter of 113 nm (± 11.4 nm, n = 50) (Fig. 1B and S2). Such size is desirable for nanoparticles preferentially retained in tumor tissues through the enhanced permeability and retention (EPR) effect [31, 32]. The dynamic light scattering (DLS) analysis (Fig. S3) demonstrates that Mn/N-HCN exhibits excellent dispersibility and stability. Anchoring of Mn^2+^ was evidenced by the increase of zeta potential of the nanoparticles from -12.97 mV to -10.6 mV (Fig. S4). The Mn content in Mn/N-HCN was quantified to be 0.75 wt% by inductively coupled plasma optical emission spectrometry (ICP-OES). Elemental mapping indicates a uniform distribution of C, N, and Mn elements (Fig. 1C). Further, individual dispersion of Mn atoms was confirmed by high-angle annular dark-field scanning transmission electron microscopy (HAADF-STEM) (Fig. 1D).

X-ray diffraction (XRD) spectrum of Mn/N-HCN exhibits two broad peaks near 22° and 43°, corresponding to graphitic carbon (Fig. 1E) [33]. It is similar to N-HCN, suggesting the absence of Mn chlorides or oxides or aggregation. X-ray photoelectron spectroscopy (XPS) survey of Mn/N-HCN also testifies the presence of Mn, N, C, and O elements (Fig. S5). The high-resolution XPS N 1 s spectrum (Fig. 1F) indicates the coexistence of pyridinic N (398.2 eV), Mn-N (399.8 eV), pyrrolic N (400.3 eV), and graphitic N (401.6 eV), suggesting that Mn is coordinated by N dopants in the graphitic carbon framework [34]. According to the high-resolution XPS Mn 2p spectrum, Mn mainly exists in the forms of Mn^2+^ (640.8 eV) and Mn^3+^ (642.2 eV) (Fig. 1G) [35]. To further determine the electronic structures and local coordination environments of Mn atoms, X-ray absorption near-edge structure (XANES) and extended X-ray absorption fine structure (EXAFS) spectroscopy at the Mn K-edge were carried out. XANES spectra in Fig. 1H show that the absorption edge of Mn K-edge is located between that of Mn foil and Mn_2_O_3_, implying that the oxidation state of Mn species lies between 0 and + 3, consistent with XPS analysis [36]. As shown by Fourier transformed (FT) κ^3^-weighted EXAFS (Fig. 1I), Mn/N-HCN presents a major scattering peak at ~ 1.60 Å corresponding to the Mn-N bond, which is distinct to Mn–Mn peak at 2.30 Å in Mn foil, Mn–O peak at 1.47 Å in Mn_2_O_3,_ and Mn–O peak at 1.40 Å in MnO [37, 38]. This observation confirms that Mn is atomically dispersed and coordinated by N. Least-squares fitting of EXAFS spectra (Fig. 1J; S6 and Table S1) suggest that Mn is coordinated by 4 N atoms with a bond length of 2.07 Å (Fig. S7) [39].

### In vitro catalytic capacity

**Fig. 2:**
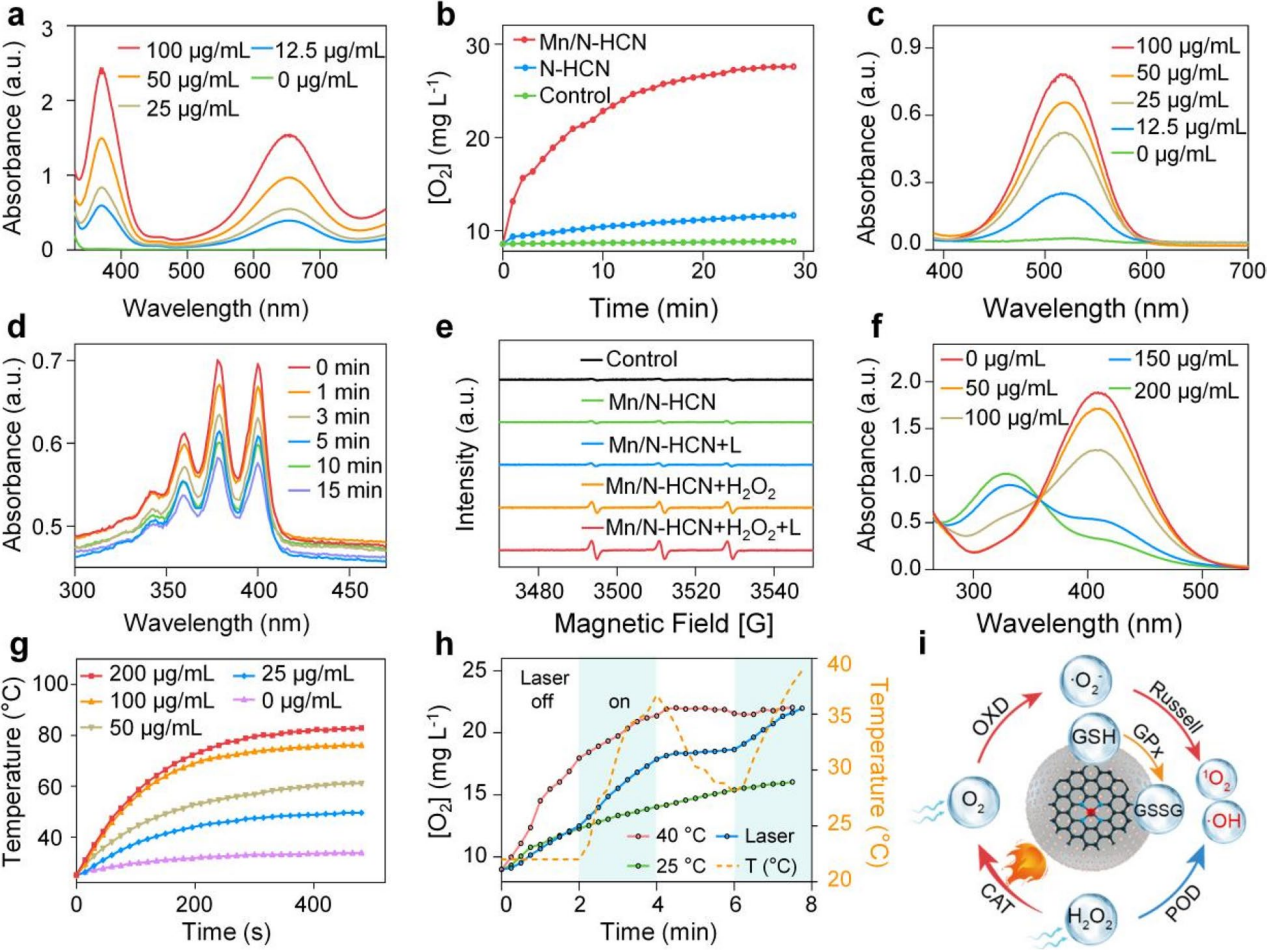
In vitro characterizations of the catalytic performance of Mn/N-HCN. **A** TMB assay for POD-mimic activity at different concentrations of Mn/N-HCN. **B** O_2_ generation from the decomposition of H_2_O_2_ (0.5 mM) due to CAT-mimic activity of N-HCN and Mn/N-HCN. **C** The •O_2_^−^ assay kit detects •O_2_^−^ production of Mn/N-HCN. The increased absorption peak at 530 nm confirms the production of •O_2_^−^. **D** ABDA assay for ^1^O_2_ generation by Mn/N-HCN. **E** ESR spectra of ^1^O_2_ at different conditions where L refers to laser irradiation and H_2_O_2_ concentration is 100 mM (The g value of Mn/N-HCN: g = 2.00665, Mn/N-HCN + L: g = 2.00666, Mn/N-HCN + H_2_O_2_: g = 2.00665, Mn/N-HCN + H_2_O_2_: g = 2.00664). **F** GSH depletion by Mn/N-HCN at different concentrations. **G** Heating curves of PBS solutions containing various amounts of Mn/N-HCN under laser irradiation. **H** O_2_ generation from the decomposition of H_2_O_2_ at 25 °C, 40 °C, or with 2 min laser on–off irradiation (while the temperature of the solution being continuously monitored). **I** Schematic illustration of coupled multi-nanocatalytic activities of Mn/N-HCN in tumor tissue. Note: The concentration of nanozyme is 100 μg/mL unless otherwise specified; the laser wavelength and power density are 1064 nm and 1 W/cm^2^, respectively

Mimicking POD activity, Mn/N-HCN can decompose H_2_O_2_ to generate highly cytotoxic •OH radical. This is testified by electron spin resonance (ESR) spectroscopy using 5,5-Dimethyl-1-pyrroline N-oxide (DMPO) as a spin trapper for •OH (Fig. S8) [40]. Using 3,3,5,5-tetramethylbenzidine (TMB) as the chromogenic substrate, which turns into blue color with strong absorption at 652 nm upon being oxidized by •OH [41], it was observed that the potency of nanozyme's POD activity was enhanced with increasing concentrations of Mn/N-HCN and H_2_O_2_ (Fig. 2A and S9) and decrease of pH (Fig. S10). The acidic microenvironment in tumor tissues is favorable for tumor-specific nanocatalytic therapy. The Michaelis–Menten constant (*K*_*m*_) and maximum initial velocity (*V*_*max*_) of Mn/N-HCN (0.078 mM and 1.99 × 10^–7^ M s^−1^, respectively) outperform natural horseradish peroxidase (0.434 mM and 1.0 × 10^–7^ M s^−1^, respectively) (Fig. S11) [42, 43]. Moreover, Mn/N-HCN exhibits markedly higher POD activity than N-HCN, implying that the catalytic activity highly depends on Mn active centers. (Fig. S12).

Also mimicking CAT activity, Mn/N-HCN catalyzes the production of O_2_ in the presence of H_2_O_2_ as detected by an oxygen meter, and it is much more potent than N-HCN (Fig. 2B). This reaction would only be slightly suppressed in the acidic tumor microenvironment (Fig. S13). Hypoxia in tumor tissues is unfavorable for the generation of ROS, thereby compromising the effectiveness of typical nanocatalytic therapy and conventional chemodynamic therapy. CAT activity of Mn/N-HCN alleviates this issue.

Mn/N-HCN demonstrates OXD activity as well, which catalyzes O_2_ to •O_2_^−^. The production of •O_2_^−^ was detected by light absorption at 530 nm using a specific superoxide anion assay kit (Fig. 2C). Additionally, ESR measurements with DMPO as the spin-trapper revealed the presence of the characteristic quadruple peaks (1:1:1:1), confirming the nanozyme's OXD activity (Fig. S14) [44]. But in tumor tissues, •O_2_^−^ can be largely scavenged by overexpressed SOD. Interestingly, it was found that Mn/N-HCN can produce highly cytotoxic ^1^O_2_ without any light exposure, as detected by the ^1^O_2_ specific probe 9,10-anthracenediyl-bis(methylene) dimalonic acid (ABDA) (Fig. 2D). The characteristic absorption peak of ABDA decreased over time, indicating continuous production of ^1^O_2_. Using 2,2,6,6-tetramethylpiperidine-N-oxyl (TEMP) as the spin-trapper, the generation of ^1^O_2_ was further evidenced by the typical 1:1:1 triplet ESR signal (Fig. 2E) [45]. Usually, nanozymes can only produce ^1^O_2_ upon laser, X-ray, or ultrasound irradiation [46]. In contrast, Mn/N-HCN requires no external energy input. Using SOD to scavenge •O_2_^−^, the production of ^1^O_2_ was completely inhibited (Fig. S15), suggesting that ^1^O_2_ was resulted from a catalytic reaction cascaded from the OXD activity of Mn/N-HCN. Conversion of •O_2_^−^ to ^1^O_2_, possibly through the Russell mechanism, has also been observed from other nanozymes [47]. In the presence of H_2_O_2_, the production of ^1^O_2_ was augmented through the cascade from CAT to OXD to Russell reaction (Fig. 2E). Taken together, Mn/N-HCN nano-multizyme can effectively catalyze H_2_O_2_ and O_2_ existing in tumor tissues to produce highly toxic •OH and ^1^O_2_ through both parallel and cascaded catalytic reactions. Glutathione (GSH), which is a highly expressed antioxidant in the tumor microenvironment, hampers the therapeutic efficiency of ROS-dependent cancer therapy [48]. Desirably, Mn/N-HCN is able to consume GSH, mimicking glutathione peroxidase (GPx). GSH can react with 5,5'-dithiobis (2-nitrobenzoic acid) (DTNB) to produce TNB. As shown in Fig. 2F and Fig. S16, the characteristic adsorption peak of TNB at 412 nm decreases with increasing Mn/N-HCN concentration and reaction time, suggesting the GPx activity of Mn/N-HCN.

Mn/N-HCN has strong light adsorption ranging from visible to NIR-II region (Fig. S17) and high photothermal conversion efficiency in a nanoparticle concentration and time-dependent manner (Fig. 2G). With 1064 nm laser irradiation at 1 W cm^−2^, the temperature of PBS solution with 50 μg/mL Mn/N-HCN increased from 23.7 to 61.1 °C within 8 min. The photothermal conversion efficiency was calculated to be 31.22% (Fig. S18) [49, 50]. Moreover, Mn/N-HCN exhibits good cycling photostability (Fig. S19). Interestingly, the CAT activity of the nanozyme can be immediately enhanced upon 1060 nm laser irradiation, correlating with the quick temperature increase due to the strong photothermal effects of the nanoparticles (Fig. 2H). Similarly, the nanozymes in the warm solution were more CAT-active.

Taken together, with five synergistically coupled nanocatalytic activities (POD, CAT, OXD, GPx, and Russell reaction), Mn/N-HCN is able to sustainably and effectively produce highly cytotoxic hydroxyl radical (•OH) and singlet oxygen (^1^O_2_) in the tumor microenvironment characterized by hypoxia and upregulated antioxidant defense (Fig. 2I). Additionally, its excellent photothermal property further promotes the production of ^1^O_2_ and allows NIR-II photoacoustic imaging to guide and monitor nanocatalytic therapy in situ.

### In vitro cellular assays

By labeling Mn/N-HCN with the green fluorescein dye Fluorescein Isothiocyanate (FITC), it was observed through confocal microscopy that the uptake of Mn/N-HCN by cells occurred in a time-dependent manner (Fig. S20). Based on cell viability tests, it was observed that Mn/N-HCN shows good biocompatibility to non-cancerous mouse fibroblast cells (L-929), but certain toxicity to mouse breast cancer cells (4T1), because H_2_O_2_ is more abundantly available in cancerous cells to be catalyzed into ROS by the nanozyme (Fig. S21). As expected, the addition of H_2_O_2_ into the culture medium and the application of laser irradiation (1064 nm) greatly enhanced the cytotoxicity. The live/dead cell staining analyses (Fig. S22) and flow cytometric measurements (Fig. S23) further confirmed the potent induction of tumor cell apoptosis by photothermally enhanced and synergized nanocatalytic therapy. Compared to the untreated control group, the Mn/N-HCN treated groups exhibited a noticeable increase in late apoptosis-induced cell death. The percentage of apoptotic cells in the Mn/N-HCN + H_2_O_2_ + laser group was significantly higher than that in both the Mn/N-HCN + H_2_O_2_ and Mn/N-HCN groups, indicating that the efficacy of nanocatalytic therapy can be greatly enhanced by leveraging the photothermal effect and H_2_O_2_ abundance in the tumor microenvironment.

**Fig. 3:**
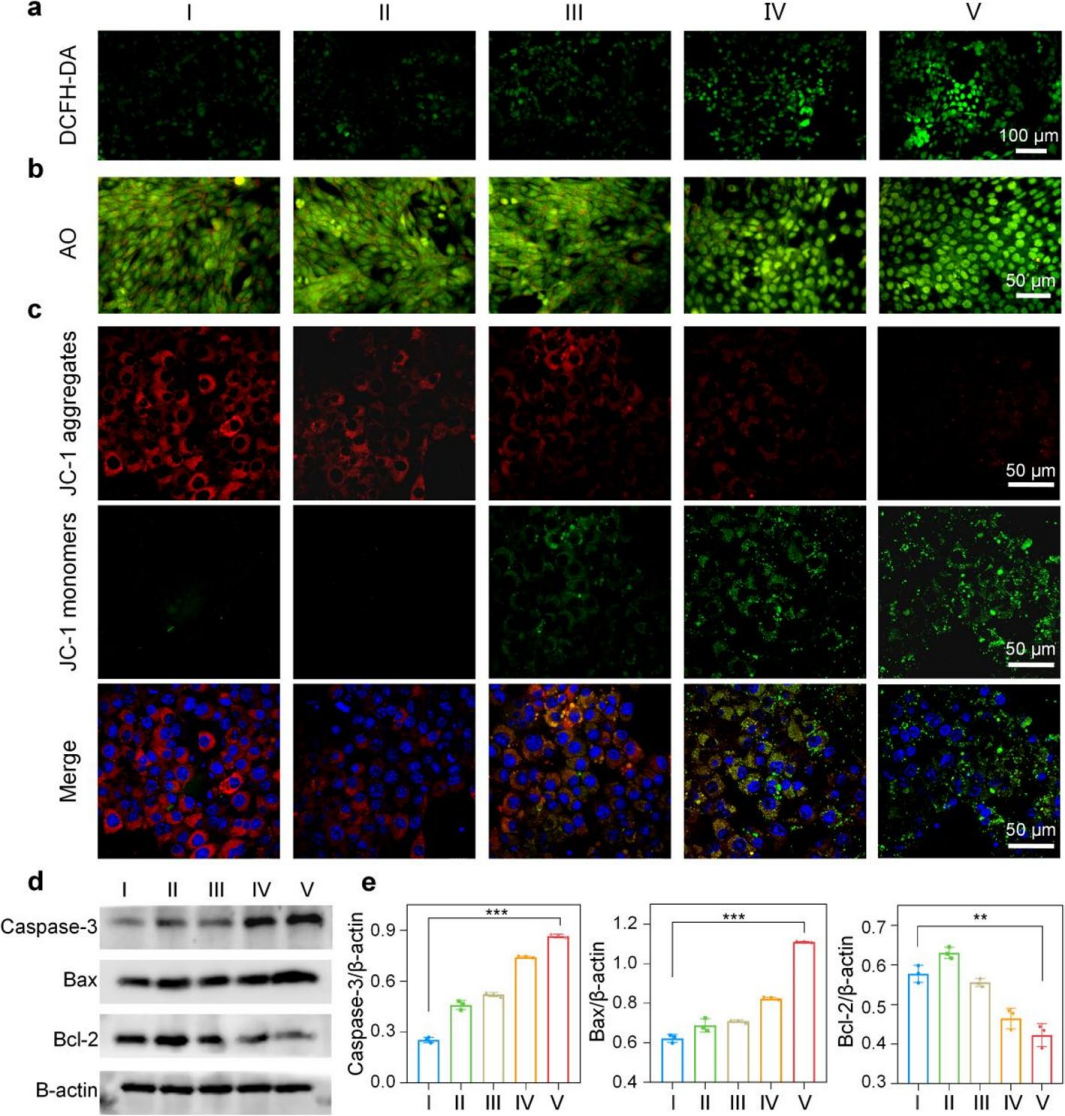
Apoptosis of cancerous 4T1 cells induced by Mn/N-HCN. **A** Intracellular ROS level. Scale bars = 50 μm. **B** AO staining. Fluorescence turns red once AO enters the cytoplasm due to compromised lysosomal integrity. Scale bars = 50 μm. **C** JC-1 analysis. At normal depolarized mitochondrial membrane potential. JC-1 molecules aggregate and emit red fluorescence. Scale bars = 50 μm. **D** Expression of apoptotic proteins revealed by immunoblotting, and (**E**) Statistic analysis normalized to actin expression. Note: different treatments: (I) Control, (II) H_2_O_2_ (100 μM), (III) Mn/N-HCN (50 μg/mL), (IV) Mn/N-HCN + H_2_O_2_, (V) Mn/N-HCN + H_2_O_2_ + 1064 nm laser (1 W/cm^2^). Statistical analysis was analyzed using Student's t-test for comparison. (*P < 0.05, **P < 0.01, ***P < 0.001, n.s. = not significant.)

The intracellular ROS level was monitored by 2,7-dichlorofluorescein diacetate (DCFH-DA), which can be oxidized by ROS to produce green fluorescent DCF. As shown in Fig. 3A, Mn/N-HCN + H_2_O_2_ + laser treatment gave the strongest fluorescence compared to the other treatments, suggesting enhanced ROS production due to increased O_2_ availability resulting from the photothermally enhanced CAT activity of Mn/N-HCN. Indeed, it was confirmed that the intracellular O_2_ was highest with Mn/N-HCN + H_2_O_2_ + laser treatment, using [Ru(dpp)_3_]Cl_2_ as the probe whose fluorescence is quenched by O_2_ (Fig. S24).

Furthermore, using acridine orange (AO) as the indicator of lysosomal integrity (Fig. 3B) and 5,5',6,6'-tetrachloro-1,1',3,3'-tetraethyl-imidacarbocyanine iodide (JC-1) as the indicator of mitochondrial membrane potential (Fig. 3C), it was concluded that photothermally-enhanced nanocatalytic therapy significantly induced apoptosis of tumor cells via lysosomal and mitochondrial disruption dependent pathways. Consistently, immunoblotting of apoptotic proteins showed that Bcl-2 was down-regulated whereas caspase-3 and Bax were up-regulated (Fig. 3D and E).

### In vivo PA imaging

**Fig. 4:**
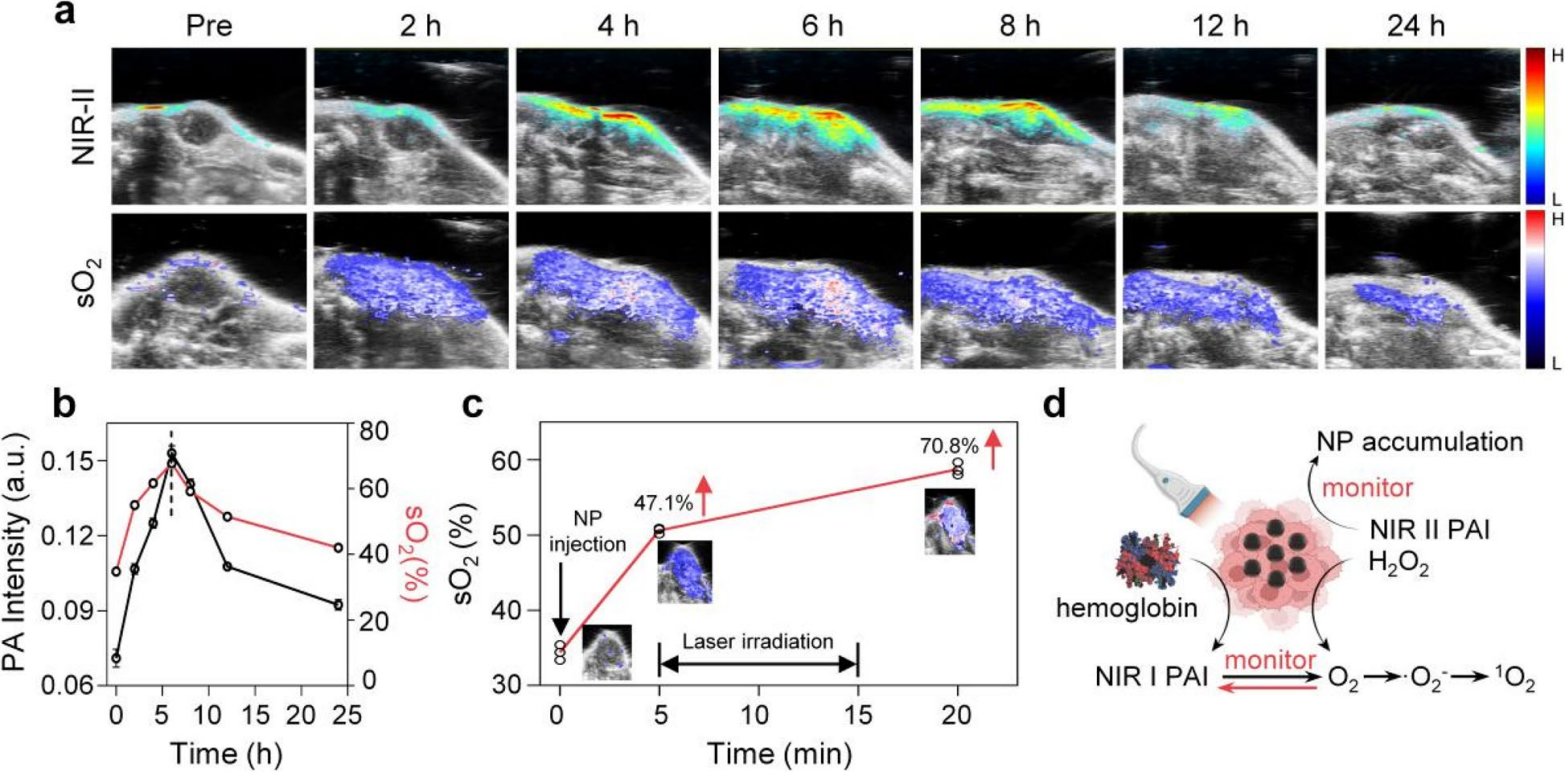
In vivo PA imaging. **A** Intra-tumor PA imaging of Mn/N-HCN and sO_2_ level after intravenous injection of Mn/N-HCN. Scale bar = 2 mm. **B** Quantification of PA intensity and sO_2_ level from (A). **C** Change of sO_2_ level after intratumoral injection of Mn/N-HCN and 1064 nm laser irradiation. **D** Schematic illustration of NIR-II/I PA monitoring

The photothermal effect of Mn/N-HCN enables photoacoustic (PA) imaging in a wide wavelength range reaching NIR-II (Fig. S25), with the signal intensity linearly proportional to the nanozyme concentration (Fig. S26). Mn/N-HCN is highly biocompatible because it doesn’t cause noticeable hemolysis even at a dose as high as 200 μg/mL (Fig. S27). Before injecting Mn/N-HCN into the tail vein, utilize Dspe-PEG-NH2 to increase the circulation time and stability. PA imaging enabled by Mn/N-HCN was then evaluated in vivo on tumor-bearing mice. As shown in Fig. 4A, the NIR-II PA signal in the tumor area became prominent 2 h after intravenous injection of Mn/N-HCN and reached the maximum at 6 h. Thereafter, NIR-II PA signals gradually decayed because of the clearance of Mn/N-HCN from tumor. This experiment demonstrates that the nanozyme preferentially accumulates in tumor tissues via the EPR effect, and 6 h after injection is the optimal treatment time (Fig. 4B). Moreover, NIR-II PA imaging revealed that nanozyme also accumulated in metabolic organs (liver, kidney, spleen) as expected but had little presence in heart and lung (Fig. S28).

Oxyhemoglobin and deoxyhemoglobin are the major endogenous NIR absorbers. Blood oxygen saturation (sO_2_) can be determined based on their strong NIR-I PA signals. As O_2_ is the key intermediate product in the catalytic cascade reaction of Mn/N-HCN, PA imaging of sO_2_ enables non-invasive real-time monitoring of the nanocatalytic therapy. After systemic intravenous administration of Mn/N-HCN, its accumulation kinetics and change of sO_2_ in tumor site are highly synchronized, demonstrating the nanozyme's ability to efficiently produce O_2_ in situ (Fig. 4B). Consistently, after direct injection of Mn/N-HCN into tumor tissue, sO_2_ level was immediately elevated by 47.1% because of the enzyme's CAT activity (Fig. 4C). It was further enhanced by 23.7% after NIR-II laser irradiation for 10 min, further confirming that the nanozyme's CAT activity can be photothermally enhanced in tumor environment. In sum, NIR PA imaging permits non-invasive, real-time, in situ monitoring of the biodistribution, tumor accumulation, and catalytic activity of the nanozyme, thereby providing guidance for the photothermally-enhanced nanocatalytic therapy and insights into its working mechanisms (Fig. 4D).

### In vivo anti-tumor assessment

**Fig. 5:**
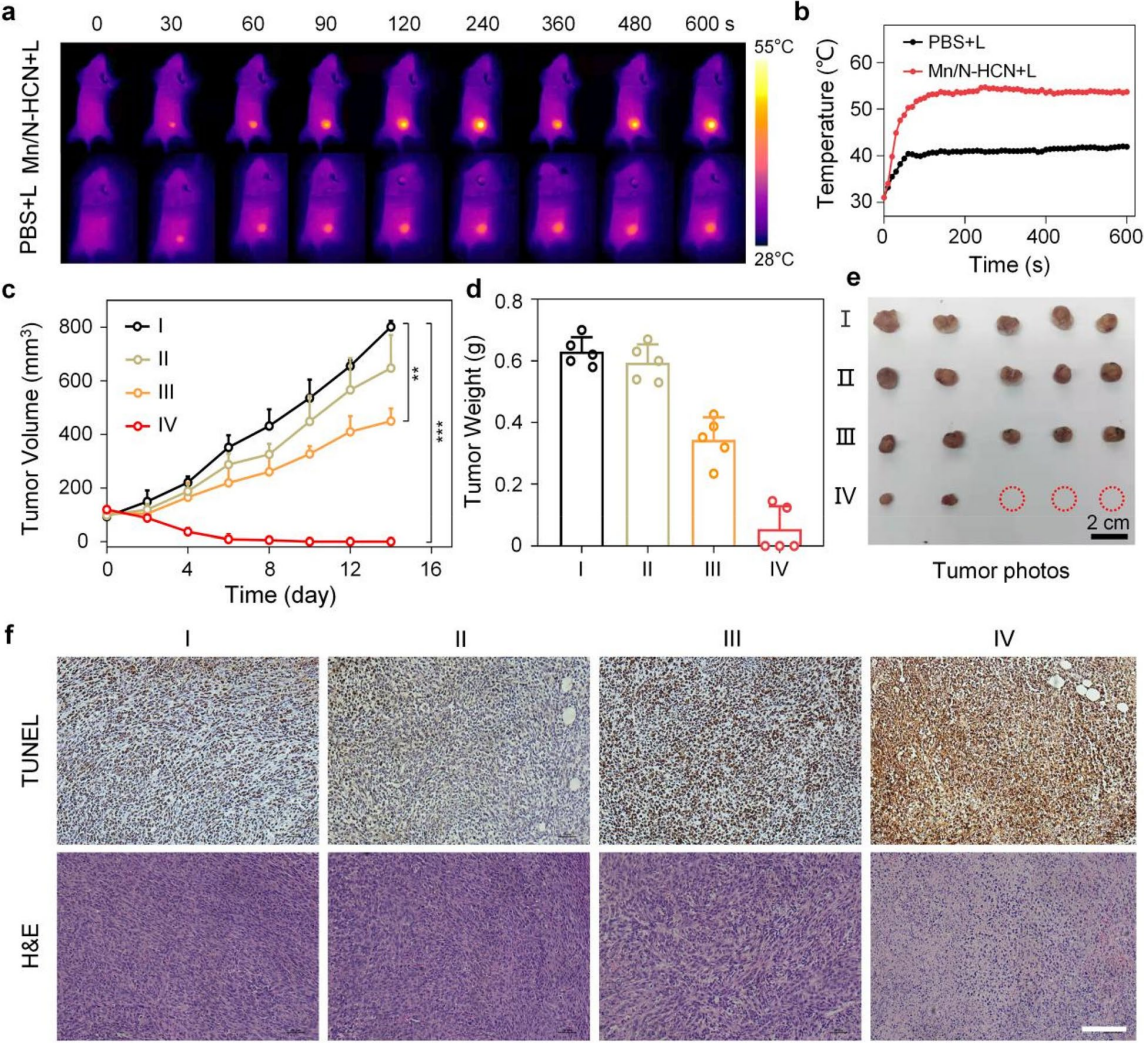
In vivo photothermally enhanced and synergized nanocatalytic therapy. **A** Infrared thermography of mice. **B** Temperature increase at tumor sites of mice. **C** Tumor growth curves. **D** Tumor weights on day 14. **E** Photographs of typically harvested tumors at day 14 after the treatments. **F** TUNEL and H&E staining of tumor tissues (Scale bar = 50 μm). In C-F), Mice were divided into 4 treatment groups as described in the main text. I: Control (intravenous injection of 200 μL PBS), II: intravenous injection of PBS + Laser irradiation (1064 nm; 1 W/cm^2^), III: intravenous injection of Mn/N-HCN (1 mg/mL, 200 μL), IV: Intravenous injection of Mn/N-HCN + Laser irradiation. (Statistical analysis was analyzed using Student's t-test for comparison. *P < 0.05, **P < 0.01, ***P < 0.001, n.s. = not significant.)

The in vivo therapeutic effects of intravenously injected Mn/N-HCN on the 4T1-bearing mice were evaluated. In the course of 2 weeks, 4 groups of mice received different treatments: I: Control (intravenous injection of 200 μL PBS), II: intravenous injection of PBS + Laser irradiation (1064 nm; 1 W/cm^2^), III: intravenous injection of Mn/N-HCN (1 mg/mL, 200 μL), IV: Intravenous injection of Mn/N-HCN + Laser irradiation. The laser was applied 6 h after injection when the Mn/N-HCN accumulation in tumor tissue reached the maximum. As presented in Fig. 5A and B, laser irradiation (1064 nm, 1 W/cm^2^) raised the temperature at tumor area to 54.0 °C within 3 min, while the temperature only increased to 40.0 °C without the nanozymes. Tumor volume was measured every 2 days. For groups I and II, the tumor volume continued to increase substantially, while Mn/N-HCN treatment (group III) significantly suppressed the tumor growth (Fig. 5C and S29). In comparison, Mn/N-HCN plus 1064 nm laser irradiation (group IV) totally inhibited tumor growth, demonstrating the high potential of photothermally enhanced nanocatalytic therapy. Finally, all mice were euthanatized, and the tumor tissues were harvested, photographed, weighed, and analyzed. The average weight of tumor tissues harvested from the mice treated with Mn/N-HCN plus 1064 nm laser is significantly lower than the other three groups (Fig. 5D and E). In comparison to the group I, the tumor growth inhibition of Mn/N-HCN treatment (group III) was 45.5%, while that of Mn/N-HCN plus 1064 nm laser treatment (group IV) was 91.5%, respectively (Fig. S30). Notably, 3 out of 5 mice in group IV achieved complete tumor elimination. The average body weights of all treated mouse groups are similar and remain steady during the treatment course, indicating that both Mn/N-HCN and laser treatment do not induce systemic toxicity (Fig. S31).

Terminal-deoxynucleotidyl transferase-mediated deoxyuridine triphosphate nick end labeling (TUNEL) immunofluorescence staining and hematoxylin and eosin (H&E) staining of the tumor tissues showed Mn/N-HCN plus laser treatment gave the most significant tumor damage with severe necrosis, nuclear condensation, and cellular atrophy (Fig. 5F). Meanwhile, no noticeable pathological abnormality was observed in major organs, such as heart, liver, spleen, lung, and kidney, indicating the long-term biosafety of Mn/N-HCN (Fig. S32). Taken together, the PA-imaging guided, photothermally enhanced and synergized nanocatalytic cancer therapy based on Mn/N-HCN nano-multizyme is safe and highly efficient.

## Discussion

Given that limited intratumoral H_2_O_2_ level and hypoxia in the TME, the parallel catalytic therapy could take advantage of multiple reactants within the tumor, such as H_2_O_2_ and O_2_, and concurrently generate a variety of ROS to enhance the tumor therapeutic efficacy. However, overexpressed GSH in the TME is another bottleneck for ROS-involved therapy. Accordingly, nanocatalytic cancer therapy is developed as an efective strategy to suppress tumor growth by producing cytotoxic ROS and eliminate GSH.

In this study, we designed a nanocatalytic Mn-based nanozymes Mn-SAE simultaneously mimics POD, CAT, and OXD to produce •O_2_^−^ and highly cytotoxic •OH. Furthermore, it catalyzes •O_2_^−^ into more cytotoxic singlet oxygen (^1^O_2_), and acts as glutathione peroxidase (GPx) to eliminate GSH thereby ensuring sustainable actions by the produced ROS.Moreover, Mn/N-HCNs possess excellent photothermal properties, which can enhance the above-mentioned catalytic process while enable PA imaging. The time-course signals of Mn/N-HCN were strongly consistent with the sO_2_ signals, demonstrating that the efficient O_2_ production in tumors is mediated by the high catalytic effect of Mn/N-HCN.

In spite of the fact that we have only preliminarily evaluated the therapeutic efficacy of Mn/N-HCN and photoacoustic in situ monitoring in a tumor mouse model, our work suggests that the combination of Mn/N-HCN with photothermal therapy can significantly inhibit tumor growth, and also that Mn/N-HCN is a promising two-region photoacoustic contrast agent. Whether it can be used in the preclinical field in the future is worth watching.

## Conclusion

Inspired by the existence of many Mn-dependent enzymes in carbon-based lifeforms, we devised a nano-multizyme with Mn-N_4_ catalytic centers atomically dispersed on a nitrogen-doped hollow carbon nanosphere (Mn/N-HCN), which is biocompatible and has a size desirable for preferential accumulation in tumor through EPR effect. Mn/N-HCN mimics the activities of four natural enzymes, including peroxidase (POD), catalase (CAT), oxidase (OXD), and glutathione peroxidase (GPx), and can also catalyze Russell reaction. Through parallel and in-series nanocatalytic reactions, the nano-multizyme can effectively produce highly cytotoxic ROS (•OH and ^1^O_2_) without the need of external energy input and simultaneously deplete upregulated antioxidant GSH. In addition, owing to high absorption in deep-tissue-penetrating NIR-II light, Mn/N-HCN provides photothermal therapy to synergize with and enhance nanocatalytic chemotherapy and enables photoacoustic imaging to guide the dual-modal treatment by revealing the biodistribution kinetics and monitoring the catalytic process in situ. This study not only demonstrates a new method for effective cancer diagnosis and therapy but also provides new insights into designing multi-functional nanozymes. In the future, we will employ photothermal-enhanced nanocatalytic strategies for treating a broader range of tumor models and monitoring catalytic process in real-time, dynamically, and quantitatively using NIR-II PA imaging.

### Supplementary Information

**Additional file 1: Figure S1**. TEM image of N-HCN. **Figure S2**. SEM image of Mn/N-HCN. **Figure S3.** The average size (A) and polydispersity index (B) of Mn/N-HCN in water at different time. **Figure S4.** Zeta potential of Mn/N-HCN. **Figure S5.** XPS spectra of Mn/N-HCN. **Figure S6.** PEXAFS fitting curves of (A) Mn_2_O_3_, (B) MnO, and (C) Mn foil in R space. **Figure S7.** The proposed Mn-N_4_-C local environment of Mn/N-HCN. **Figure S8.** ESR spectra of •OH with characteristic quartet signal (1:2:2:1) of DMPO-OH. **Figure S9.** Time-course absorbance changes of TMB with the addition of different concentrations of H_2_O_2_. **Figure S10.** TMB assay for measuring POD-mimic activity of Mn/N-HCN from pH 4.6 to pH. **Figure S11.** (A) Michaelis-Menten kinetics curves for the POD-mimic activity of Mn/N-HCN, (B) corresponding Lineweaver-Burk plots. **Figure S12**. TMB assay for measuring POD-mimic activity of Mn/N-HCN. **Figure S13.** pH-dependent CAT-mimic activity of Mn/N-HCN. **Figure S14.** ESR spectra of •O_2_^-^ with characteristic peaks 1:1:1:1 of DMPO-OOH. **Figure S15.** ABDA assay for ^1^O_2_ generation by Mn/N-HCN, in the presence of SOD. **Figure S16.** GSH depletion by Mn/N-HCN during different times. **Figure S17.** UV-Vis-NIR spectra of N-HCN and Mn/N-HCN. **Figure S18.** Calculation of photothermal conversion efficiency of Mn/N-HCN (100 µg/mL) under irradiation with 1064 nm laser (1 W/cm^2^). Red curve: Photothermal profile of Mn/N-HCN irradiated for 7 min. Blue curve: followed by nature cooling. Linear time data versus -ln(θ) obtained from the cooling period. **Figure S19.** Temperature of Mn/N-HCN containing PBS solution subjecting to on/off cycling of laser irradiation. **Figure S20.** Uptake of FITC-label Mn/N-HCNs by 4T1 cells at different times. **Figure S21.** (A) Cell viability of L929 cells was detected by the CCK-8 method after 12 h-treatment of Mn/N-HCN at different concentrations. (B) Cell viability of 4T1 cells after 12 h-treatment of Mn/N-HCN at different concentrations and conditions. **Figure S22.** Live/dead cell assay. The green signal from Calcein-AM indicates live cells and the red signal from PI indicates dead cells. Scale bars=50 μm. **Figure S23.** Flow cytometric measurement on cells co-stained with Annexin V-FITC and PI. **Figure S24.** Intracellular O_2_ generation using [Ru(dpp)_3_]Cl_2_ as a probe: (I) Control; (II) Mn/N-HCN+H_2_O_2_ (100 μM); (III) Mn/N-HCN+H_2_O_2_+L. Scale bars=50μm. **Figure S25.** The photoacoustic spectra of the Mn/N-HCN. The wavelength range is from 1200 nm to 1600 nm. **Figure S26.** (A) NIR-II (1200 nm) PA signal of Mn/N-HCN at different concentrations in PBS. (B) PA intensity to nanoparticle concentration with a linear fitting. **Figure S27.** In vitro haemocompatibility assay of Mn/N-HCN with different concentrations ranges from 0 to 200 µg/mL. **Figure S28.** Biodistribution of Mn/N-HCN in various organs after 6 h post-injection. **Figure S29.** Photographs of tumors at 1, 7, and 14 day after different treatments (I Control, II PBS+laser, III Mn/N-HCN, IV Mn/N-HCN+Laser). **Figure S30.** Tumor Inhibition Rate after different treatments (I Control, II PBS+laser, III Mn/N-HCNs, IV Mn/N-HCN+Laser). **Figure S31.** Body weight during 14 days (I Control, II PBS+laser, III Mn/N-HCNs, IV Mn/N-HCN+Laser). **Figure S32.** H&E staining of major organs (heart, liver, spleen, lung, and kidney) dissected from the BALB/c mice with different treated groups (I Control, II PBS+laser, III Mn/N-HCN, IV Mn/N-HCN+Laser) for post 14 days injection (Scale bar: 50 μm). **Table S1.** Parameters of EXAFS fits for Mn foil, MnO, Mn_2_O_3_, and Mn/N-HCN.

## Data Availability

The datasets used and/or analyzed during the current study are available from the corresponding author on reasonable request.

## Abbreviations

POD: Peroxidase
CAT: Catalase
OXD: Oxidase
GPx: Glutathione peroxidase
GSH: Glutathione
•OH: Hydroxyl radical
^1^O_2_: Singlet oxygen
•O_2_^−^: Superoxide radicals
PTT: Photothermal therapy
NIR-II: Near-Infrared-II
PA: Photoacoustic
ROS: Reactive oxygen species
SAEs: Single-atom nanozymes
SOD: Superoxide dismutase
TMB: 3,5,3',5’-Tetramethylbenzidine
ABDA: 9,10-Anthracenediyl-bis(methylene) dimalonic acid
ESR: Electron paramagnetic resonance
TEMP: 2,2,6,6-Tetramethylpiperidine-N-oxyl
DMPO: 5,5-Dimethyl-1-pyrroline N-oxide
DTNB: 5,5'-Dithiobis 2-nitrobenzoic acid
DCFH-DA: 2,7-Dichlorodi-hydrofuorescein diacetate
AO: Acridine orange
sO_2_: Saturated vascular O_2_
H&E: Hematoxylin-eosin staining
