# Supplementary material for: Mn-single-atom nano-multizyme enabled NIR-II photoacoustically monitored, photothermally enhanced ROS storm for combined cancer therapy
Source: Biomater Res. 2023 Dec 4;27:125. doi: 10.1186/s40824-023-00464-w (PMC10694968; doi:10.1186/s40824-023-00464-w)
Supplement: Supplementary file 1 — Additional file 1: Figure S1. TEM image of N-HCN. Figure S2. SEM image of Mn/N-HCN. Figure S3. The average size (A) and polydispersity index (B) of Mn/N-HCN in water at different time. Figure S4. Zeta potential of Mn/N-HCN. Figure S5. XPS spectra of Mn/N-HCN. Figure S6. PEXAFS fitting curves of (A) Mn2O3, (B) MnO, and (C) Mn foil in R space. Figure S7. The proposed Mn-N4-C local environment of Mn/N-HCN. Figure S8. ESR spectra of •OH with characteristic quartet signal (1:2:2:1) of DMPO-OH. Figure S9. Time-course absorbance changes of TMB with the addition of different concentrations of H2O2. Figure S10. TMB assay for measuring POD-mimic activity of Mn/N-HCN from pH 4.6 to pH. Figure S11. (A) Michaelis-Menten kinetics curves for the POD-mimic activity of Mn/N-HCN, (B) corresponding Lineweaver-Burk plots. Figure S12. TMB assay for measuring POD-mimic activity of Mn/N-HCN. Figure S13. pH-dependent CAT-mimic activity of Mn/N-HCN. Figure S14. ESR spectra of •O2- with characteristic peaks 1:1:1:1 of DMPO-OOH. Figure S15. ABDA assay for 1O2 generation by Mn/N-HCN, in the presence of SOD. Figure S16. GSH depletion by Mn/N-HCN during different times. Figure S17. UV-Vis-NIR spectra of N-HCN and Mn/N-HCN. Figure S18. Calculation of photothermal conversion efficiency of Mn/N-HCN (100 µg/mL) under irradiation with 1064 nm laser (1 W/cm2). Red curve: Photothermal profile of Mn/N-HCN irradiated for 7 min. Blue curve: followed by nature cooling. Linear time data versus -ln(θ) obtained from the cooling period. Figure S19. Temperature of Mn/N-HCN containing PBS solution subjecting to on/off cycling of laser irradiation. Figure S20. Uptake of FITC-label Mn/N-HCNs by 4T1 cells at different times. Figure S21. (A) Cell viability of L929 cells was detected by the CCK-8 method after 12 h-treatment of Mn/N-HCN at different concentrations. (B) Cell viability of 4T1 cells after 12 h-treatment of Mn/N-HCN at different concentrations and conditions. Figure S22. Live/dead cell assay. The green si [file 40824_2023_464_MOESM1_ESM.docx]

Supplementary Material

**Mn-Single-Atom Nano-multizyme Enabled NIR-II Photoacoustically Monitored, Photothermally Enhanced ROS Storm for Combined Cancer Therapy**

Xiaozhe Wang ^1,2 #^, Xiaofeng Ren ^3,#^, Jie Yang ^4^, Zican Zhao ^4^, Xiaoyu Zhang ^1,2^, Fan Yang ^3^, Zheye Zhang ^5^, Peng Chen ^5 *^, Liping Li ^1, 3 *^, Ruiping Zhang ^1*^

^1^ The Radiology Department of Shanxi Provincial People's Hospital, Five Hospital of Shanxi Medical University, Taiyuan, 030001, China

^2^ College of Medical Imaging, Shanxi Medical University, Taiyuan, 030001, China

^3^ Department of Biochemistry and Molecular Biology, School of Basic Medical Sciences, Shanxi Medical University, Taiyuan 030001, China

^4^ Third Hospital of Shanxi Medical University, Shanxi Bethune Hospital, Shanxi Academy of Medical Sciences, Taiyuan 030032, China

^5^ School of Chemistry, Chemical Engineering and Biotechnology, Lee Kong Chian School of Medicine, Institute for Digital Molecular Analytics and Science, Nanyang Technological University, 62 Nanyang Drive, 637459, Singapore.

^#^ These authors contributed equally to this study.

^*^ Corresponding Author: Peng Chen (chenpeng@ntu.edu.sg), Liping Li (liliping_8103@163.com), Ruiping Zhang (zrp_7142@sxmu.edu.cn)

**Figure S1** TEM image of N-HCN.

**Figure S2** SEM image of Mn/N-HCN.


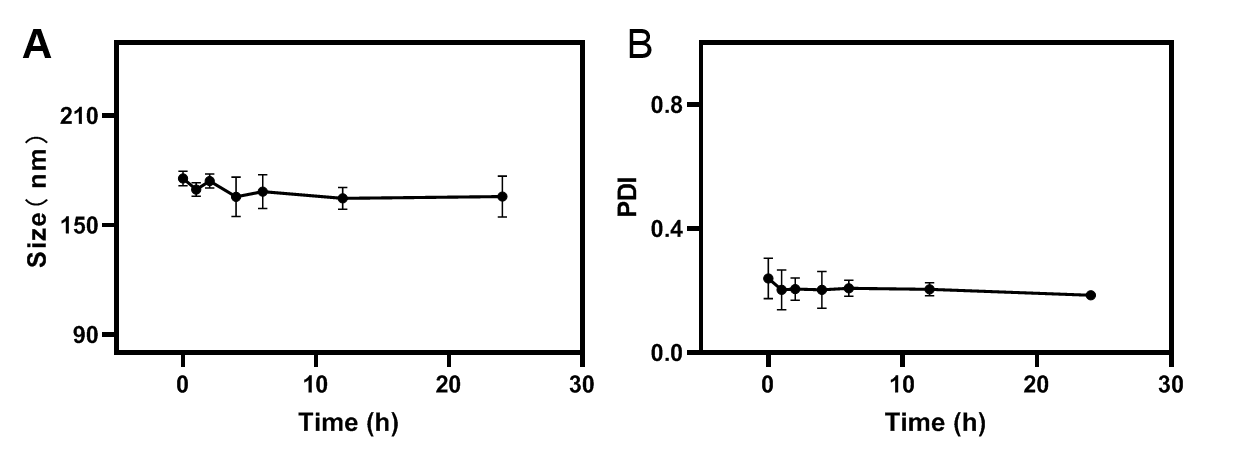


**Figure S3** The average size (A) and polydispersity index (B) of Mn/N-HCN in water at different time.


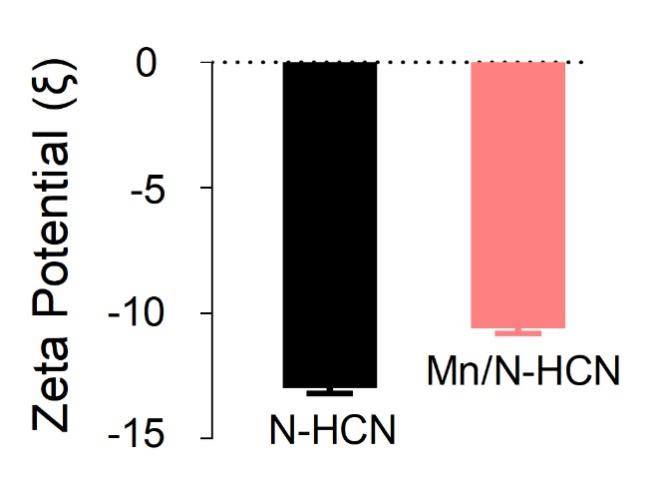


**Figure S4** Zeta potential of Mn/N-HCN.

**Figure S5** XPS spectra of Mn/N-HCN.


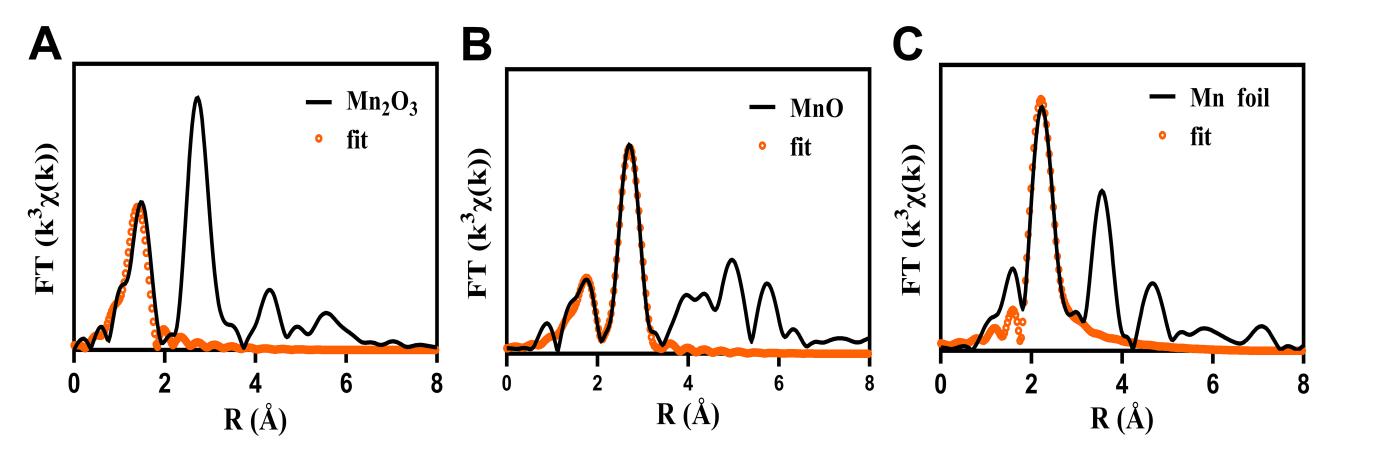


**Figure S6** EXAFS fitting curves of (A) Mn_2_O_3,_ (B) MnO, and (C) Mn foil in R space.


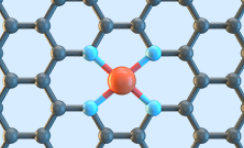


**Figure S7** The proposed Mn-N_4_-C local environment of Mn/N-HCN.


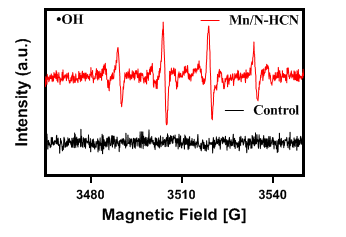


**Figure S8** ESR spectra of •OH with characteristic quartet signal (1:2:2:1) of DMPO-OH (g=2.00650).

**Figure S9** Time-course absorbance changes of TMB with the addition of different concentrations of H_2_O_2_.

**Figure S10** TMB assay for measuring POD-mimic activity of Mn/N-HCN from pH 4.6 to pH 7.4.


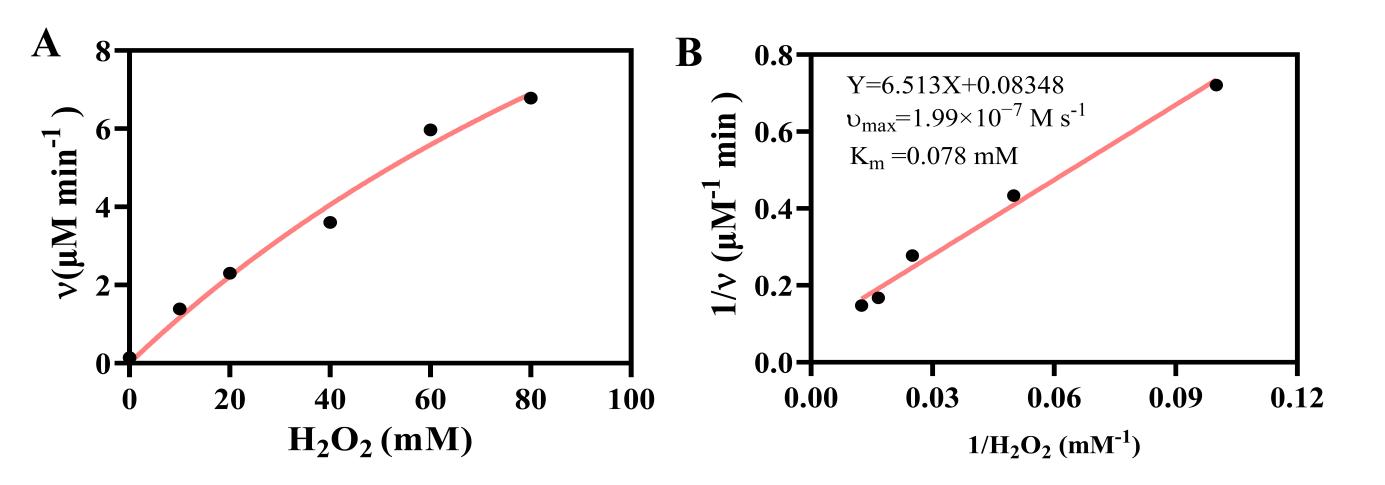


**Figure S11** (A) Michaelis-Menten kinetics curves for the POD-mimic activity of Mn/N-HCN, (B) corresponding Lineweaver-Burk plots.

**Figure S12** TMB assay for measuring POD-mimic activity of Mn/N-HCN.

**Figure S13** pH-dependent CAT-mimic activity of Mn/N-HCN.


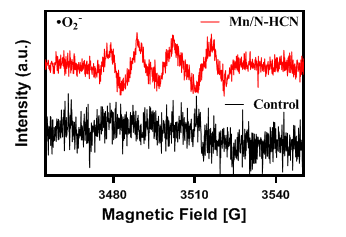


**Figure S14** ESR spectra of •O_2_^-^ with characteristic peaks 1:1:1:1 of DMPO-OOH (g=2.00740).

**Figure S15** ABDA assay for ^1^O_2_ generation by Mn/N-HCN, in the presence of SOD.

**Figure S16** GSH depletion by Mn/N-HCN during different times.

**Figure S17** UV-Vis-NIR spectra of N-HCN and Mn/N-HCN.

**Figure S18** Calculation of photothermal conversion efficiency of Mn/N-HCN (100 µg/mL) under irradiation with 1064 nm laser (1 W/cm^2^). Red curve: Photothermal profile of Mn/N-HCN irradiated for 7 min. Blue curve: followed by nature cooling. Linear time data versus -ln(θ) obtained from the cooling period.

**Figure S19** Temperature of Mn/N-HCN containing PBS solution subjecting to on/off cycling of laser irradiation.


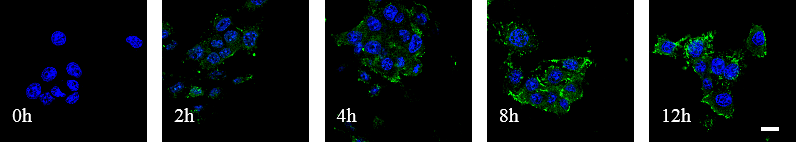


Figure S20 Uptake of FITC-label Mn/N-HCNs by 4T1 cells at different times. Green fluorescence corresponds to FITC, blue fluorescence corresponds to DAPI. Scale bars=20 μm.


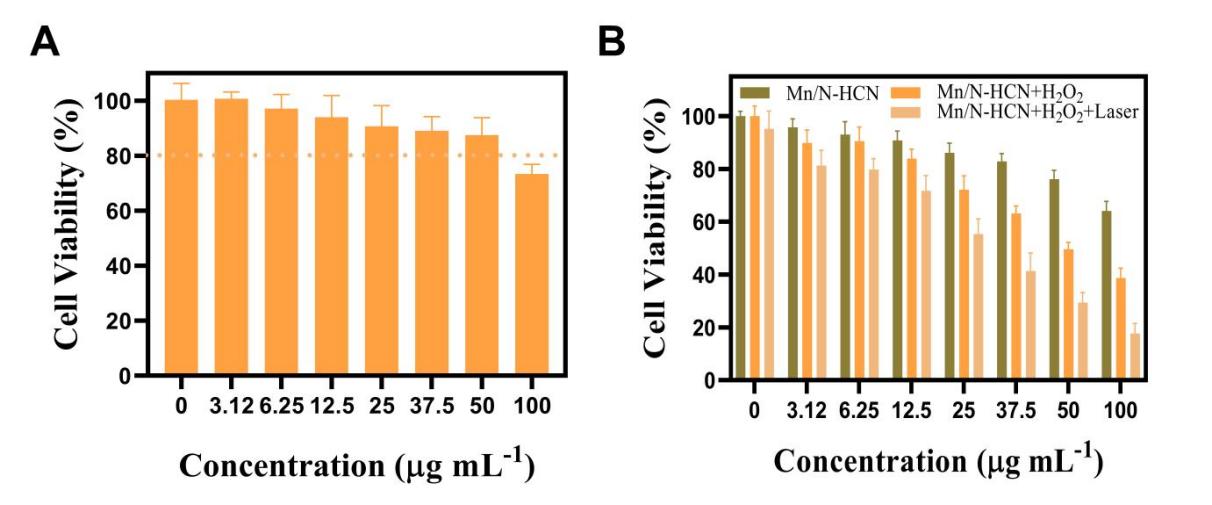


**Figure S21** **(**A) Cell viability of L929 cells was detected by the CCK-8 method after 12 h-treatment of Mn/N-HCN at different concentrations. (B) Cell viability of 4T1 cells after 12 h-treatment of Mn/N-HCN at different concentrations and conditions.


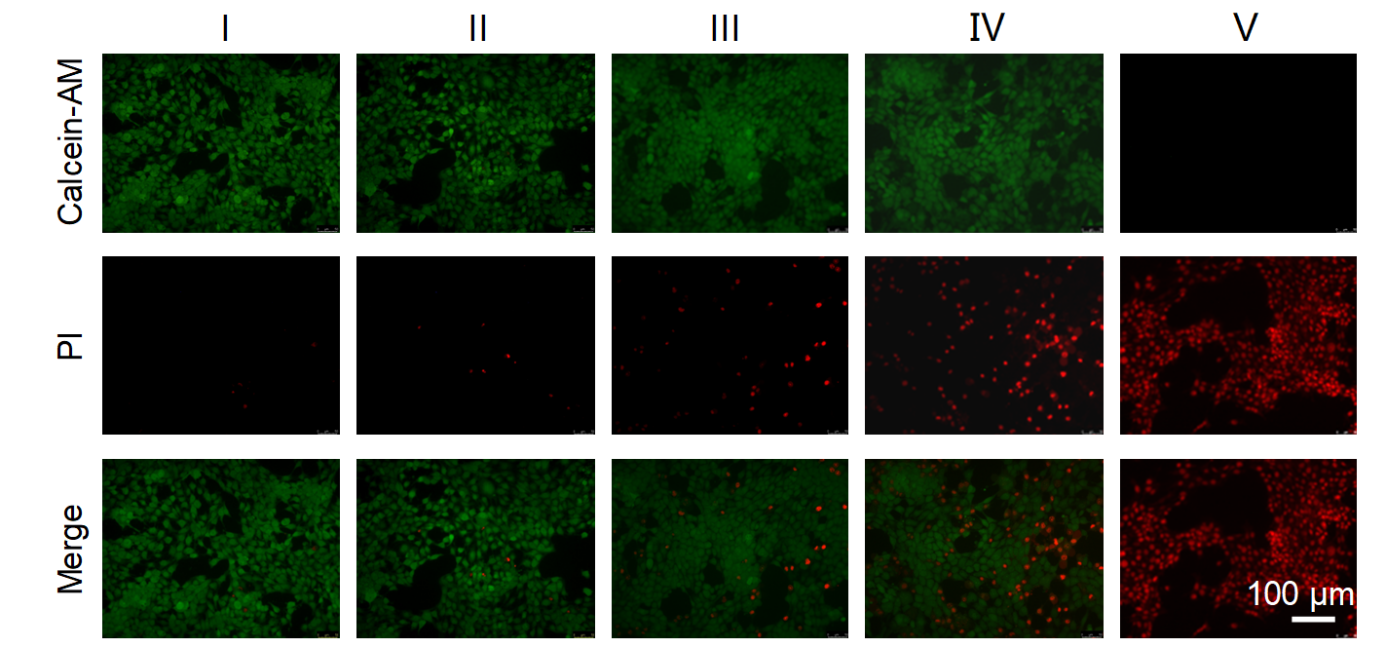


**Figure S22** Live/dead cell assay. The green signal from Calcein-AM indicates live cells and the red signal from PI indicates dead cells. Scale bars=50 μm.

**Figure S23** Flow cytometric measurement on cells co-stained with Annexin V-FITC and PI.


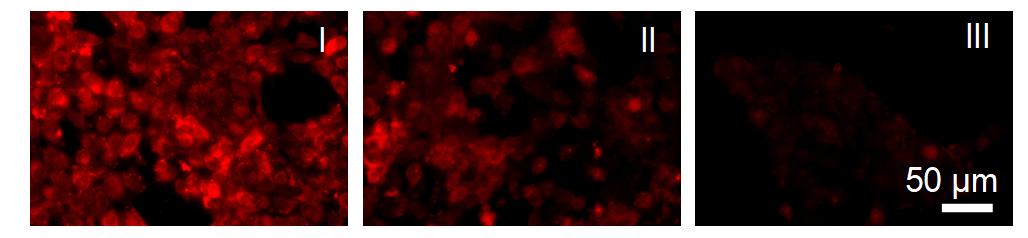


**Figure S24** Intracellular O_2_ generation using [Ru(dpp)_3_]Cl_2_ as a probe: (I) Control; (II) Mn/N-HCN+H_2_O_2_ (100 μM); (III ) Mn/N-HCN+H_2_O_2_+L. Scale bars=50μm.

**Figure S25** The photoacoustic spectra of the Mn/N-HCN. The wavelength range is from 1200 nm to 1600 nm.


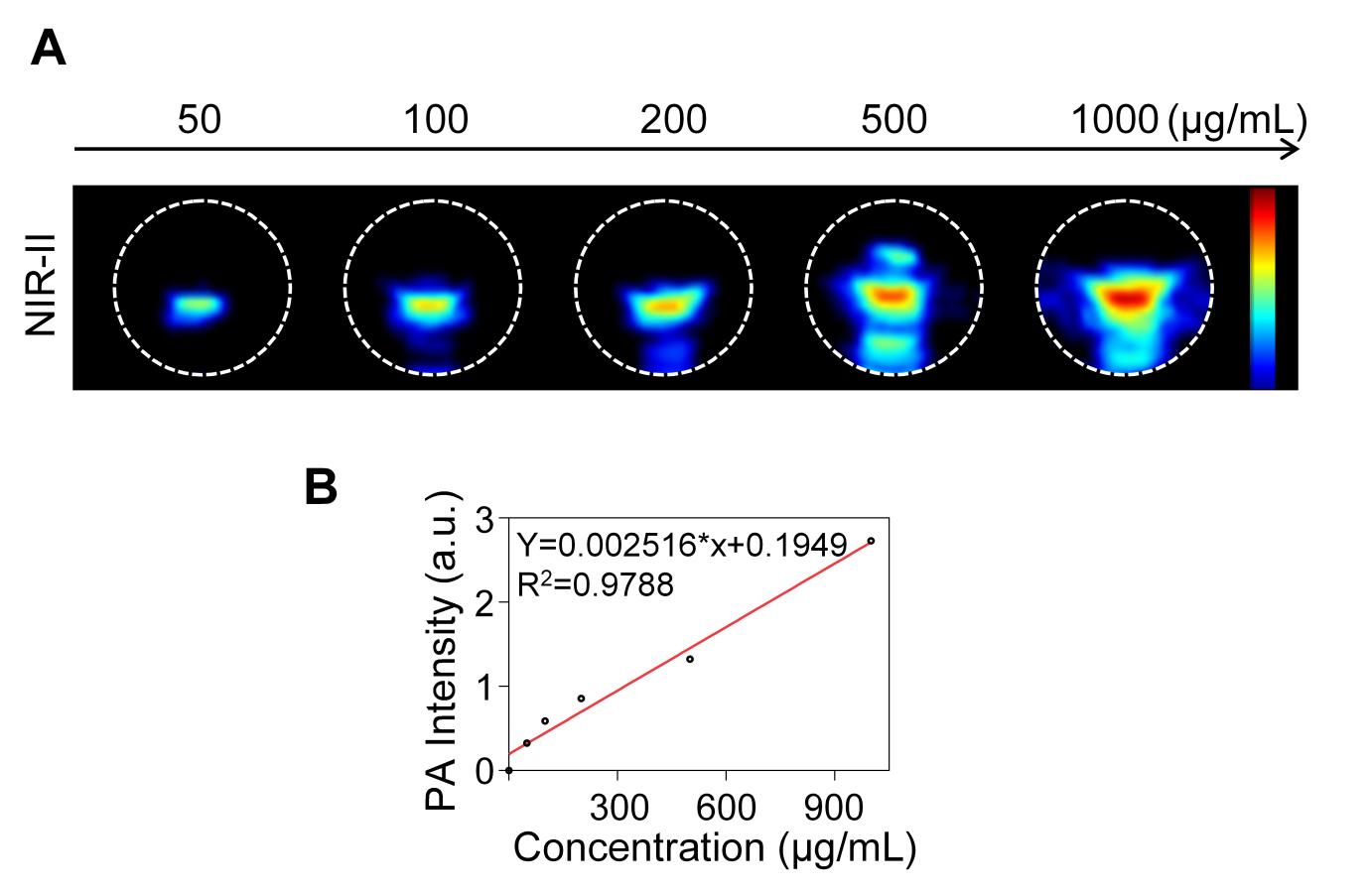


**Figure S26** (A) NIR-II (1200 nm) PA signal of Mn/N-HCN at different concentrations in PBS. (B) PA intensity to nanoparticle concentration with a linear fitting.

**Figure S27** In vitro haemocompatibility assay of Mn/N-HCN with different concentrations ranges from 0 to 200 µg/mL.

**Figure S28** Biodistribution of Mn/N-HCN in various organs after 6 h post-injection.

**Figure S29** Photographs of tumors at 1, 7, and 14 day after different treatments (I Control, II PBS+laser, III Mn/N-HCN, IV Mn/N-HCN+Laser).

**Figure S30** Tumor Inhibition Rate after different treatments (I Control, II PBS+laser, III Mn/N-HCNs, IV Mn/N-HCN+Laser).

**Figure S31** Body weight during 14 days (I Control, II PBS+laser, III Mn/N-HCNs, IV Mn/N-HCN+Laser).


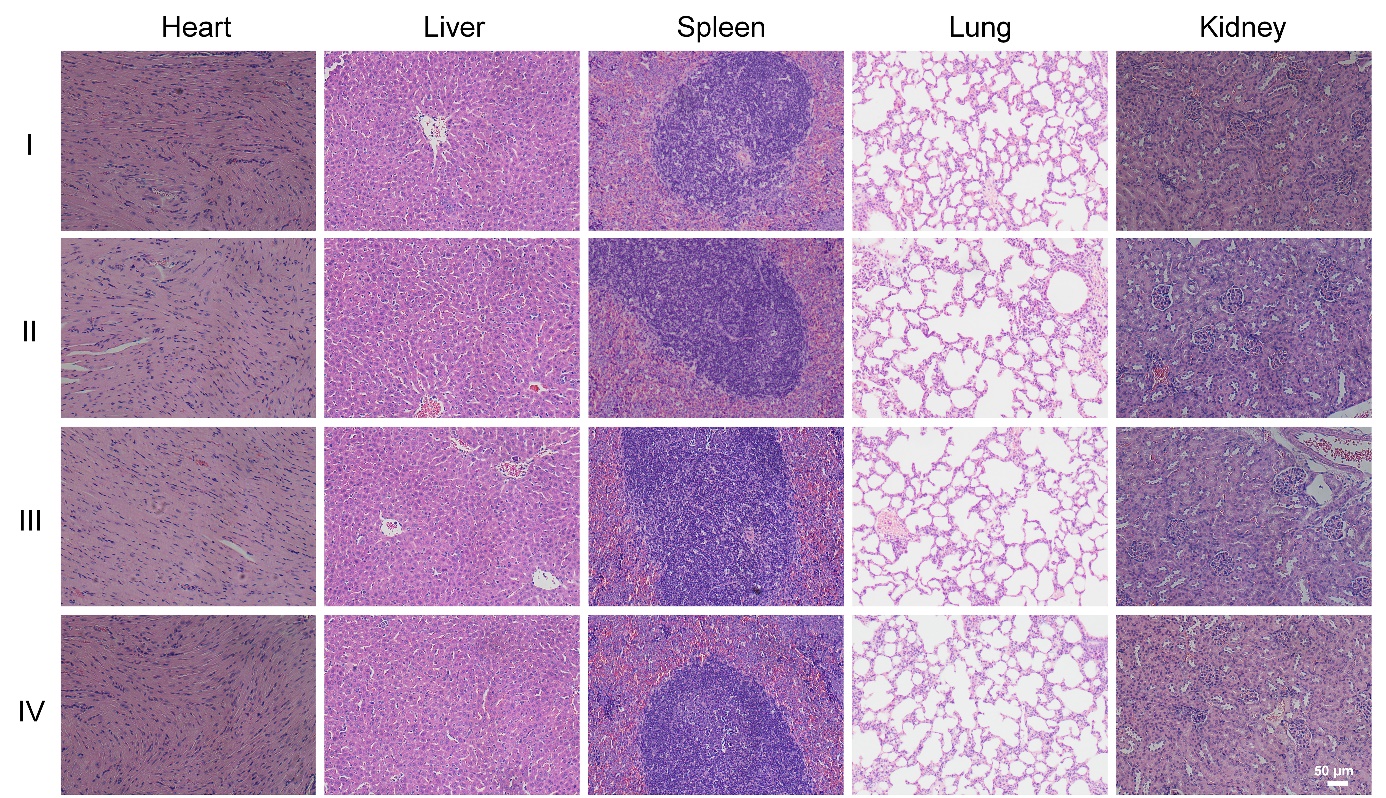


**Figure S32** H&E staining of major organs (heart, liver, spleen, lung, and kidney) dissected from the BALB/c mice with different treated groups (I Control, II PBS+laser, III Mn/N-HCN, IV Mn/N-HCN+Laser) for post 14 days injection. (Scale bar: 50 μm)

**Table S1** Parameters of EXAFS fits for Mn foil, MnO, Mn_2_O_3_, and Mn/N-HCN.

| Samples | Path | *R*(Å) | *CN* | σ^2^(Å^2^∙10^-3^) | *∆E^0^*(eV) | R factor(%) |
| --- | --- | --- | --- | --- | --- | --- |
| Mn foil | Mn-Mn | 2.67±0.04 | 12 | 0.01 | -8.81 | 0.30 |
| MnO | Mn-O | 2.25±0.03 | 6 | 10.24 | 2.4 | 0.005 |
|  | Mn-Mn | 3.18±0.05 | 12 | 9.28 | -2.1 |  |
| Mn_2_O_3_ | Mn-O | 1.97±0.10 | 2 | 7.1 | -11.76 | 0.67 |
| Mn/N-HCN (this work) | Mn-N | 2.07±0.09 | 4.4 | 1.9 | -1.0 | 0.012 |
|  | Mn-O | 2.25±0.07 | 0.04 | 1.3 | 1.6 |  |

CN, coordination number; R, the distance between the absorber and backscatter atoms; σ^2^, Debye-Waller factor to account for thermal and structural disorders; ΔE^0^, inner potential correction; R factor (%) indicates the goodness of the fit. Error bounds (accuracies) that characterize the structural parameters obtained by EXAFS spectroscopy were estimated as CN ± 20%; R ± 1%; σ^2^ ± 20%; ΔE^0^ ± 20%.
